# Supplementary material for: Disentangling the drivers of heterogeneity in SARS-CoV-2 transmission from data on viral load and daily contact rates
Source: PLoS Comput Biol. 2026 Aug 28;22(8):e1014715. doi: 10.1371/journal.pcbi.1014715 (PMC13552949; doi:10.1371/journal.pcbi.1014715)
Supplement: Supplementary file 1 [file pcbi.1014715.s001.pdf]

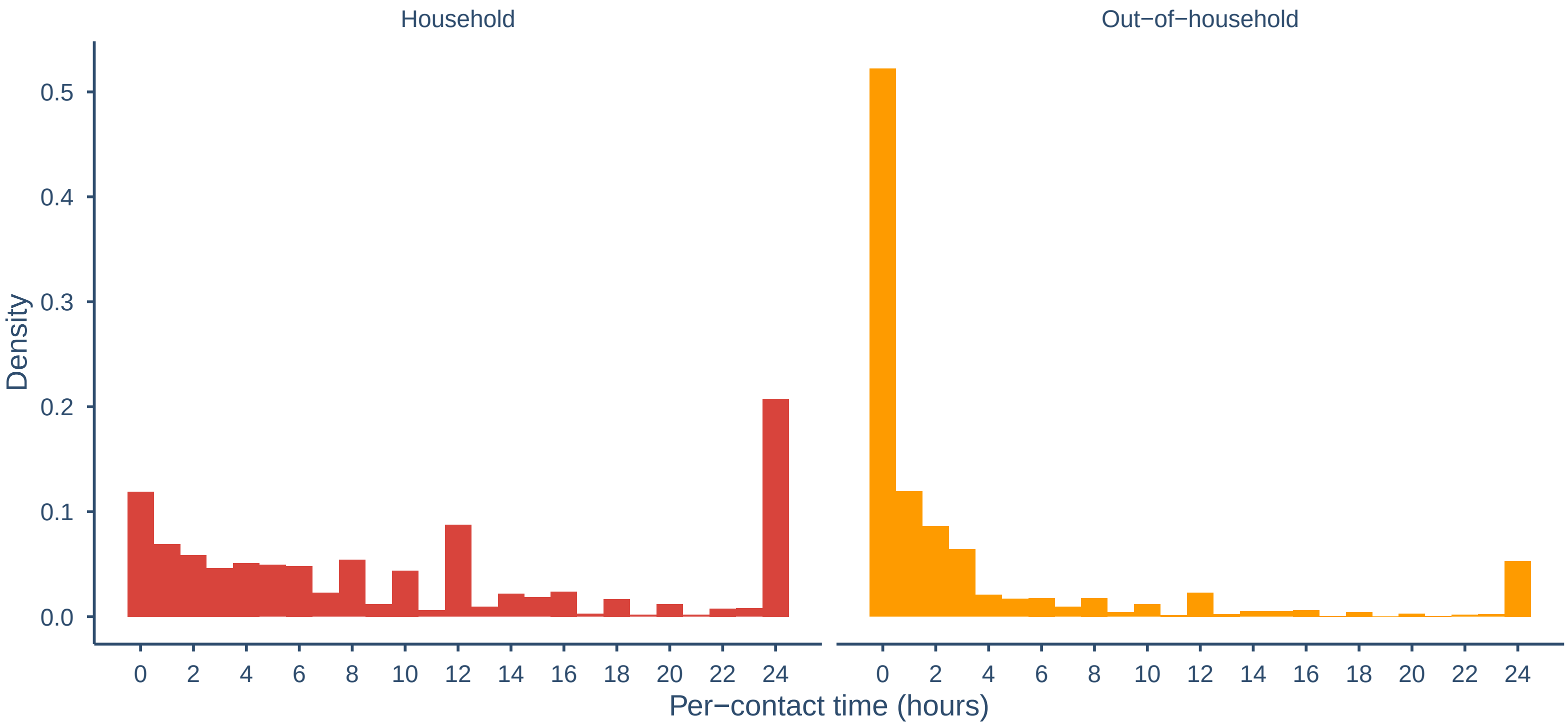

Household
Out−of−household
Density
0.0
0.1
0.2
0.3
0.4
0.5
0
2
4
6
8
10
12
14
16
18
20
22
24
Per−contact time (hours)
