# Supplementary material for: Disentangling the drivers of heterogeneity in SARS-CoV-2 transmission from data on viral load and daily contact rates
Source: PLoS Comput Biol. 2026 Aug 28;22(8):e1014715. doi: 10.1371/journal.pcbi.1014715 (PMC13552949; doi:10.1371/journal.pcbi.1014715)
Supplement: Supplementary file 2 [file pcbi.1014715.s002.pdf]

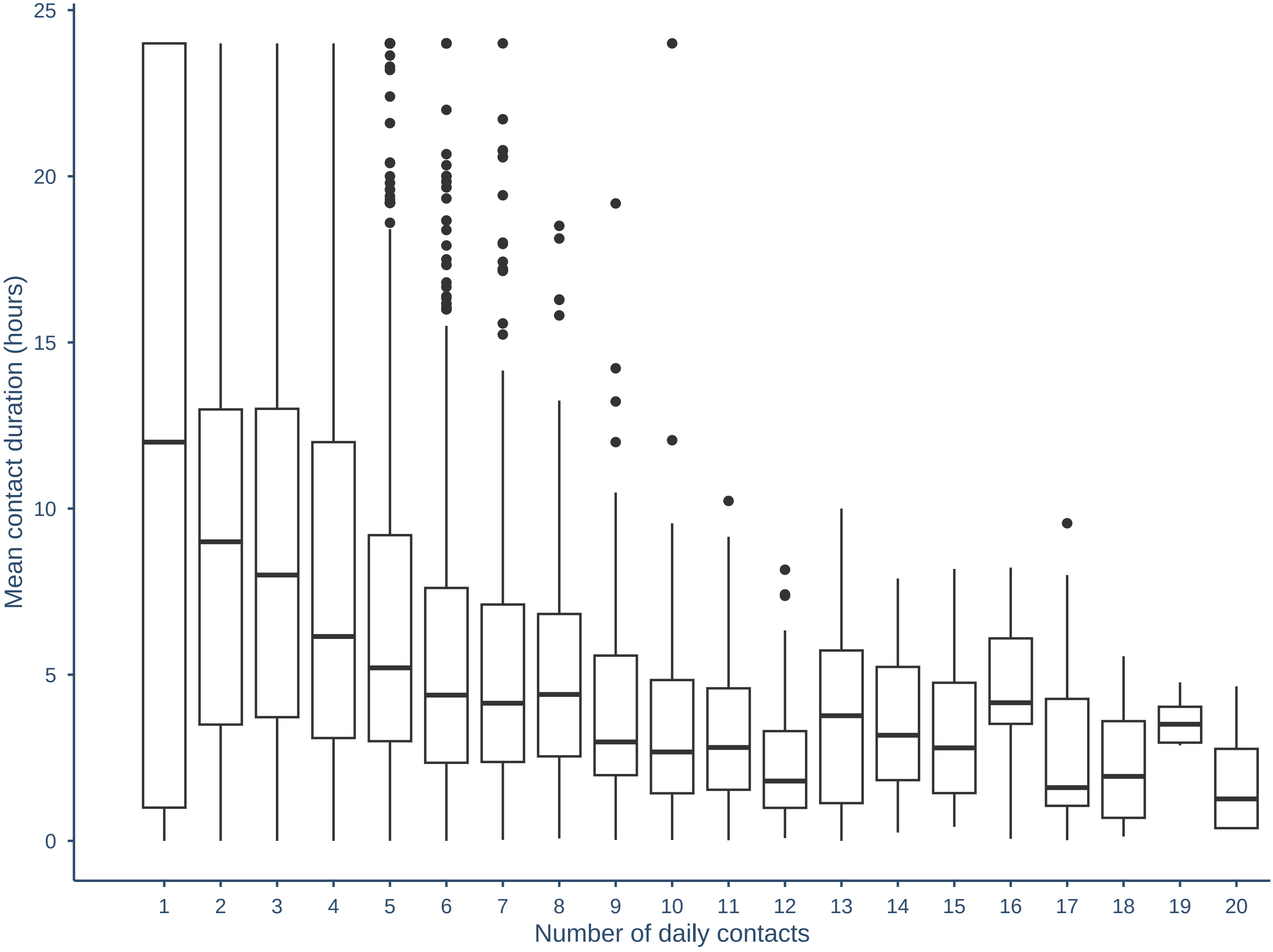

Mean contact duration (hours)
0
5
10
15
20
25
1
2
3
4
5
6
7
8
9
10
11
12
13
14
15
16
17
18
19
20
Number of daily contacts
