# Supplementary material for: Disentangling the drivers of heterogeneity in SARS-CoV-2 transmission from data on viral load and daily contact rates
Source: PLoS Comput Biol. 2026 Aug 28;22(8):e1014715. doi: 10.1371/journal.pcbi.1014715 (PMC13552949; doi:10.1371/journal.pcbi.1014715)
Supplement: Supplementary file 5 [file pcbi.1014715.s005.pdf]

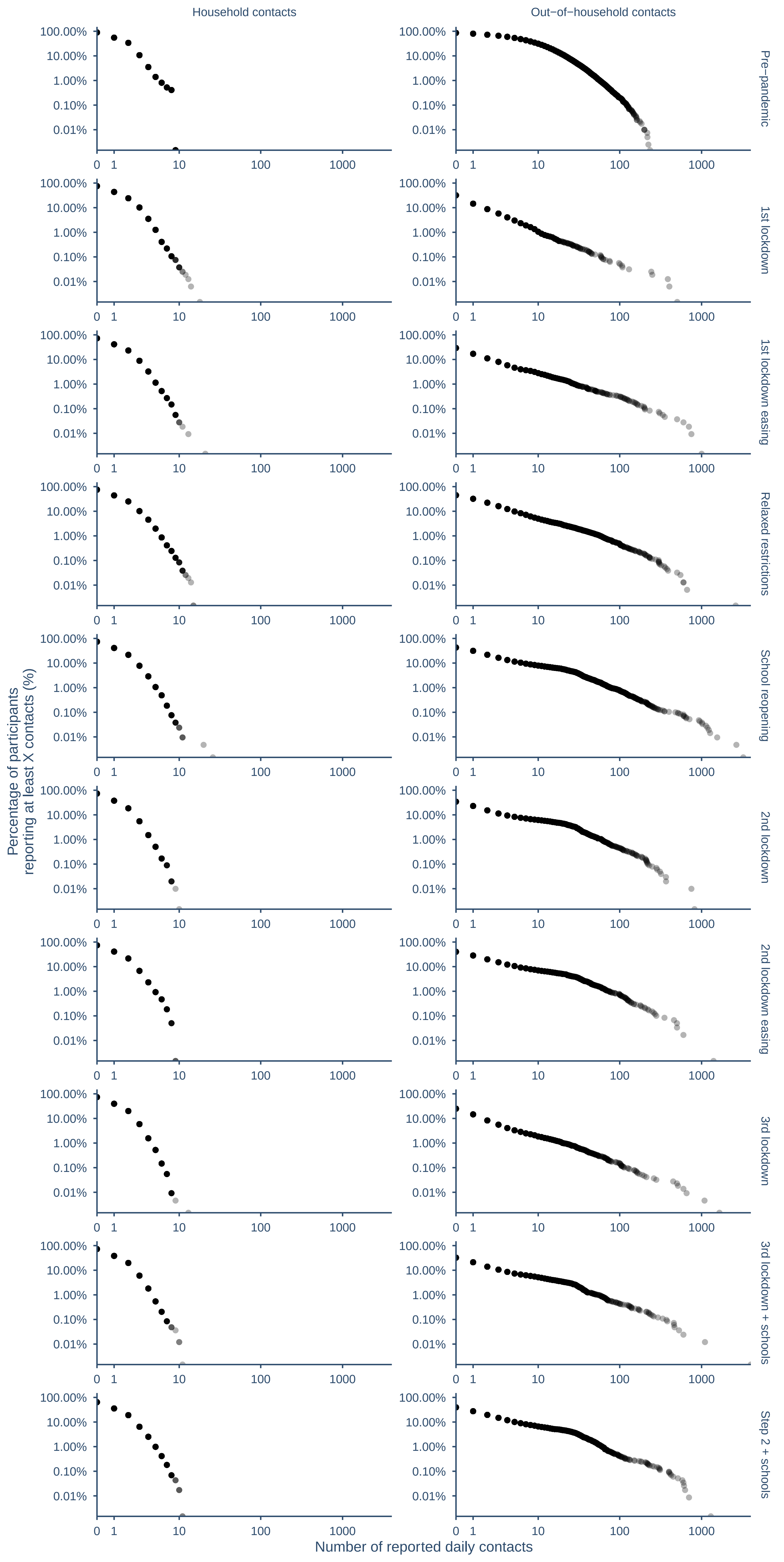

Household contacts
Out−of−household contacts
Pre−pandemic
1st lockdown
1st lockdown easing
Relaxed restrictions
School reopening
2nd lockdown
2nd lockdown easing
3rd lockdown
3rd lockdown + schools
Step 2 + schools
100.00%
10.00%
1.00%
0.10%
0.01%
0
1
10
100
1000
Percentage of participants reporting at least X contacts (%)
Number of reported daily contacts
