# Supplementary material for: Disentangling the drivers of heterogeneity in SARS-CoV-2 transmission from data on viral load and daily contact rates
Source: PLoS Comput Biol. 2026 Aug 28;22(8):e1014715. doi: 10.1371/journal.pcbi.1014715 (PMC13552949; doi:10.1371/journal.pcbi.1014715)
Supplement: Supplementary file 6 [file pcbi.1014715.s006.pdf]

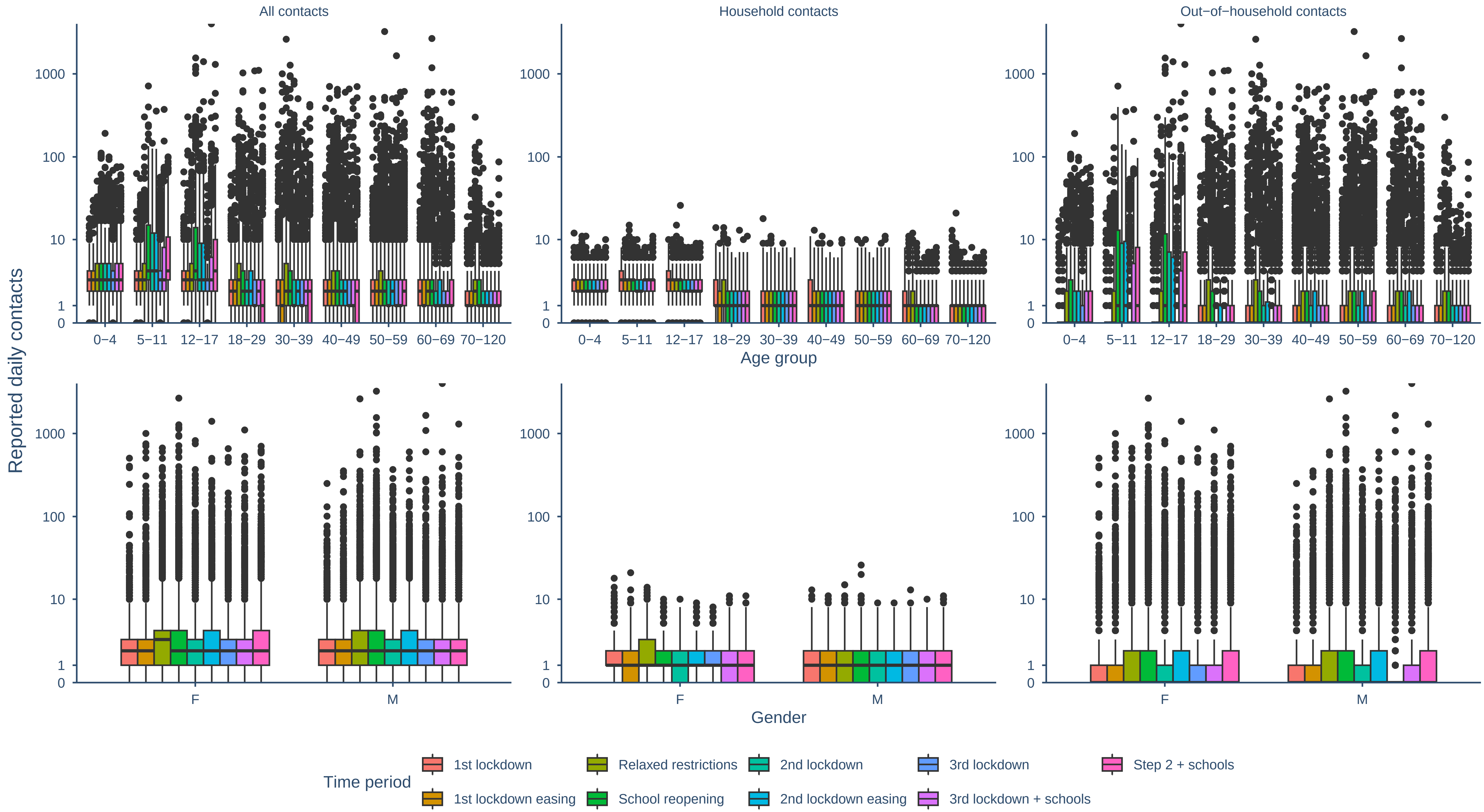
All contacts
Household contacts
Out−of−household contacts
Reported daily contacts
0
1
10
100
1000
0−4
5−11
12−17
18−29
30−39
40−49
50−59
60−69
70−120
Age group
F
M
Gender
Time period
1st lockdown
1st lockdown easing
Relaxed restrictions
School reopening
2nd lockdown
2nd lockdown easing
3rd lockdown
3rd lockdown + schools
Step 2 + schools
