# Supplementary material for: Disentangling the drivers of heterogeneity in SARS-CoV-2 transmission from data on viral load and daily contact rates
Source: PLoS Comput Biol. 2026 Aug 28;22(8):e1014715. doi: 10.1371/journal.pcbi.1014715 (PMC13552949; doi:10.1371/journal.pcbi.1014715)
Supplement: Supplementary file 7 [file pcbi.1014715.s007.pdf]

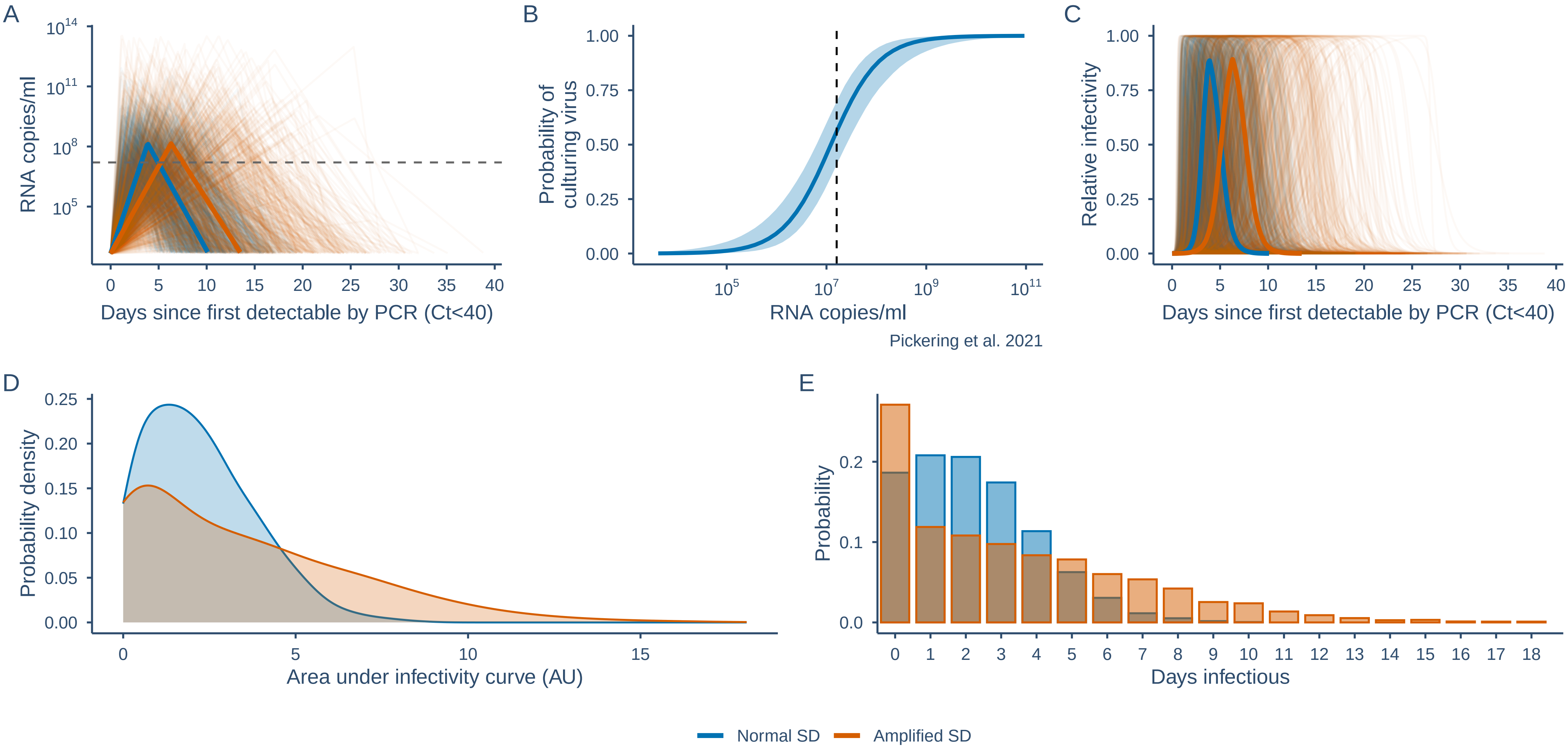

A
RNA copies/ml
$10^{14}$
$10^{11}$
$10^{8}$
$10^{5}$
0 5 10 15 20 25 30 35 40
Days since first detectable by PCR (Ct<40)
B
Probability of culturing virus
1.00 0.75 0.50 0.25 0.00
$10^{5}$ $10^{7}$ $10^{9}$ $10^{11}$
RNA copies/ml
Pickering et al. 2021
C
Relative infectivity
1.00 0.75 0.50 0.25 0.00
0 5 10 15 20 25 30 35 40
Days since first detectable by PCR (Ct<40)
D
Probability density
0.25 0.20 0.15 0.10 0.05 0.00
0 5 10 15
Area under infectivity curve (AU)
E
Probability
0.2 0.1 0.0
0 1 2 3 4 5 6 7 8 9 10 11 12 13 14 15 16 17 18
Days infectious
Normal SD
Amplified SD
