# Supplementary material for: Disentangling the drivers of heterogeneity in SARS-CoV-2 transmission from data on viral load and daily contact rates
Source: PLoS Comput Biol. 2026 Aug 28;22(8):e1014715. doi: 10.1371/journal.pcbi.1014715 (PMC13552949; doi:10.1371/journal.pcbi.1014715)
Supplement: Supplementary file 8 [file pcbi.1014715.s008.pdf]

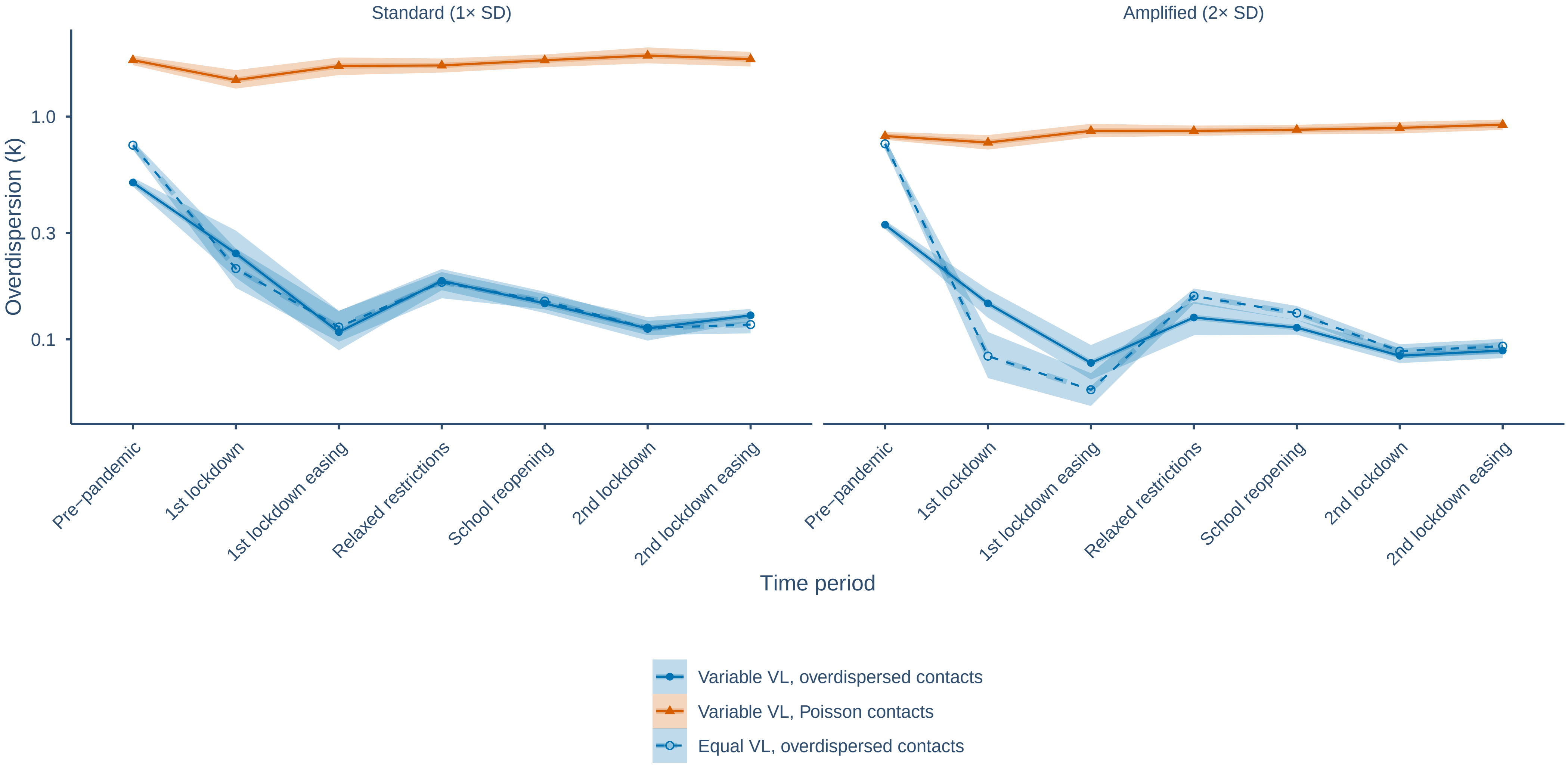

Left: VL parameters as estimated from Kissler et al.
Right: SDs of peak Ct, proliferation, and clearance all doubled.
