# Supplementary material for: Disentangling the drivers of heterogeneity in SARS-CoV-2 transmission from data on viral load and daily contact rates
Source: PLoS Comput Biol. 2026 Aug 28;22(8):e1014715. doi: 10.1371/journal.pcbi.1014715 (PMC13552949; doi:10.1371/journal.pcbi.1014715)
Supplement: Supplementary file 9 [file pcbi.1014715.s009.pdf]

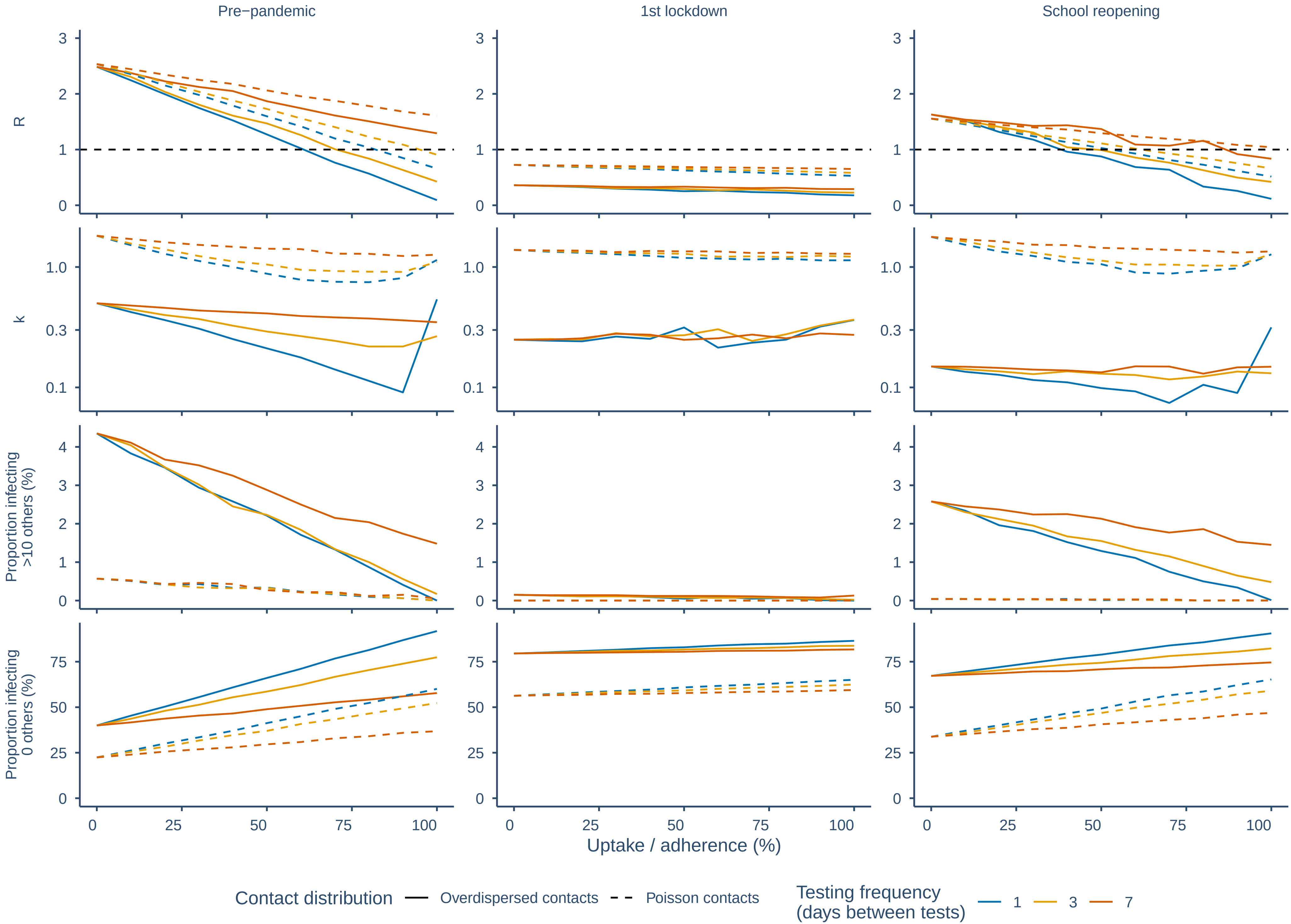

Pre−pandemic
1st lockdown
School reopening
R
k
Proportion infecting >10 others (%)
Proportion infecting 0 others (%)
Uptake / adherence (%)
Contact distribution
Overdispersed contacts
Poisson contacts
Testing frequency (days between tests)
1
3
7
