# Supplementary material for: Disentangling the drivers of heterogeneity in SARS-CoV-2 transmission from data on viral load and daily contact rates
Source: PLoS Comput Biol. 2026 Aug 28;22(8):e1014715. doi: 10.1371/journal.pcbi.1014715 (PMC13552949; doi:10.1371/journal.pcbi.1014715)
Supplement: Supplementary file 10 [file pcbi.1014715.s013.pdf]

School of Mathematical Sciences
Lancaster University
Balrigg
Lancaster
LA1 4YR

The Editors,
PLOS Computational Biology

28th April 2026

Dear Editors,

Thank you for reviewing our manuscript and requesting we submit a revised version. We would like to thank the reviewers for their careful review of the manuscript and helpful comments and suggestions. The paper has been thoroughly revised in light of these comments – please see below for detailed responses. We believe we have addressed all of the reviewers' comments and that this has substantially improved the manuscript.

Kind regards,

Lloyd Chapman and Billy Quilty
l.chapman4@lancaster.ac.uk
billy.quilty@lshtm.ac.uk

Additional Editor Comments:

The reviewers feel that your question and approach are interesting but raise several points that should be addressed for your manuscript to meet the requirements of publication in PLOS Computational Biology.

The key points raised include:

1. Justifying or acknowledging design choices regarding heterogeneities in duration of infection (R1), and number and duration of contacts (R2), and potential biases/limitations of data sources (for both viral kinetics and contacts) (R1; R3)

2. Clarity of methods (R1; R2; R3) – see note on manuscript structure below

3. Variation in epidemiological parameters over duration of analysis (ie, emergence of new variants and increasing population exposure) (R1)

4. Overstating conclusions (R2; R3)

5. R3 raises a pertinent point re the design of studies seeking to assess the importance of different drivers of an observed phenomenon, that warrants discussion

Note re manuscript structure: two reviewers commented specifically on the structure of the manuscript, and the infelicity of having Methods appear after Results for some types of study. Note that while suggested the default organization in the submission guidelines place Methods after Results, this is not a strict requirement. The submission guidelines also state: “To provide flexibility, however, authors are also able to include the Materials and Methods section before the Results section or before the Discussion section.” This may be appropriate here.

We thank you for highlighting these key points raised by the reviewers. We have addressed all of these in our revisions – please see below for detailed responses – and believe that this has considerably strengthened the manuscript.

Journal Requirements:

1) Please ensure that the CRediT author contributions listed for every co-author are completed accurately and in full.
At this stage, the following Authors/Authors require contributions: Billy J Quilty, Lloyd A. C. Chapman, James D Munday, Kerry L M Wong, Amy Gimma, Suzanne Pickering, Stuart J D Neil, Rui Pedro Galão, W. John Edmunds, Chris I Jarvis, and Adam J Kucharski. Please ensure that the full contributions of each author are acknowledged in the "Add/Edit/Remove Authors" section of our submission form.
The list of CRediT author contributions may be found here:
https://journals.plos.org/ploscompbiol/s/authorship#loc-author-contributions

2) We ask that a manuscript source file is provided at Revision. Please upload your manuscript file as a .doc, .docx, .rtf or .tex. If you are providing a .tex file, please upload it under the item type u2018LaTeX Source Fileu2019 and leave your .pdf version as the item type u2018Manuscriptu2019.

3) Please upload all main figures as separate Figure files in .tif or .eps format. For more information about how to convert and format your figure files please see our guidelines:
https://journals.plos.org/ploscompbiol/s/figures

4) We have noticed that you have uploaded Supporting Information files, but you have not included a list of legends. Please add a full list of legends for your Supporting Information files after the references list.

Reviewers' comments:

All line numbers below refer to the track changes version of the manuscript "Manuscript_track_changes.docx" viewed in the desktop version of Word with "Show Revisions in Balloons" under "Mark-up Options".

Reviewer's Responses to Questions

Comments to the Authors:
Please note here if the review is uploaded as an attachment.

Reviewer #1: Summary

The authors study the drivers of superspreading events in the case of SARS-CoV-2 (i.e. the fact that a minority of individuals causes the majority of the infections). More precisely, they with to determine whether these are caused by variations in virus load among individuals or whether they originate from behavioural variations. For this, they lean on existing databases regarding within-host temporal variations in virus load and temporal variations in the number of reported contacts. They use existing studies to perform numerical simulations with a simplified epidemiological model, e.g. without epidemiological feedbacks. The complement this model to explore counterfactual scenarios where later-flow testing can prevent transmission from individuals with high virus load. They interpret their results in the context of the literature, while highlighting potential limitations of their approach.

Review

I have three major concerns about the manuscript. First, although the model is designed to be simple, I find some of its assumptions overly simplistic. Second, the literature does not seem to be up-to-date for such rapidly moving field (there are few references from 2023 and hardly any from 2024). Third, I am unsure that the manuscript's strengths (public health insights) align with our journal's scope (mathematical and computational biology).

We thank the reviewer for raising their concerns and have tried to address each of them as detailed below.

1) The model

The space devoted to the model is limited, even when accounting for the Supplementary Materials, but the modelling appears to be simple. In essence, the authors assume all the infections are independent (there is no depletion of susceptible hosts) and, for each infection initiated at time t, draw a number of household contacts and a number of non-household contacts (both of which depend on t) using empirical data. Every day of infection, they then perform a Bernoulli trial to determine if any of the contacts were infected or not.

We have now moved the information about the model that was in the Supporting Information into the main text and added further detail (pp 5-10 lines 142-275). It was not in fact the case that there was no depletion of susceptible hosts in the model, as household members can only be infected once. However, this was not explained in the Methods, so we have now added the following on p8 lines 218-220:

"While we modelled infection events for household members on each day of the index case's infection, we only counted the first infection that occurred for each household member. In other words, household members could only be infected once."

1.1 My main critique about the model is that the authors tend to force the heterogeneity in virus kinetics to play in terms of the magnitude of the peak virus load. Another possibility, which I think would increase the window of transmission, would be to investigate the duration of the infection. As shown in UK data, in some infections, this duration can exceed 60 days. Although this may only represent a small proportion of the population (0.1 to 0.5 %), it might be compensated by the length of the transmission period (at least 6 times that of a traditional generation time). Such long infections are

not present in the cohort analysed but could likely be included using cohorts that are more representative of the general population (in fact, a sensitivity analysis would be required to show that the exact shape of the virus kinetics matters on the result).

We agree with the reviewer that much longer durations of shedding for some infections could have an impact on the contribution of viral load heterogeneity to heterogeneity in the secondary infection distribution. To investigate this, we have conducted a sensitivity analysis of the impact of greater variation in the shape of the viral load trajectories on variation in numbers of secondary infections. We reran our analysis with viral load trajectories simulated from truncated normal distributions for the proliferation time, clearance time, and Ct peak parameters with double the standard deviations of those used in the main analysis. The results of this analysis are presented in Fig. S5 and discussed in lines 631-637 p18 and show that while greater variation in peak viral load and shedding duration increase the contribution of viral load heterogeneity to heterogeneity in secondary infections, the dominant contribution is still from heterogeneity in contact numbers.

1.2 The temporal component of the model seems very limited. For example, the authors assume that the generation time (i.e. the time between two infections) is constant, which is at odds with the evolution of the Alpha variant or behavioural changes. In the Discussion, they specify that this is why this model was run only over 2020. This seems to be at odds with Figures 1, 3, and 4. If the authors wish to restrict their analysis to 2020, they should do it consistently throughout the manuscript. Conversely, if they analyse results up to mid-2021, they need to account for these temporal variations.

The figure showing the contact distributions from the BBC Pandemic and CoMix contact surveys (now Figure 2, previously Figure 1) shows data for the nine time periods from the CoMix survey up to May 2021. Figures 4 and 5 (old Figures 3 and 4) only show results from 2020, up to the easing of the 2nd lockdown, as was mentioned in the text of the Results section. We feel it is helpful to present information on the contact distributions from CoMix for all nine time periods, including the three in 2021, to give a sense of how contact distributions evolved over the pandemic, and since it is consistent with the presentation of the CoMix data in other publications (Gimma et al. 2022). We have now moved the statement about restricting the secondary infection distribution analysis to 2020 to the Methods under “Reconstruction of secondary infection distribution”:

“We restricted our analysis to the first six time periods of the CoMix survey in 2020 prior to the widespread emergence of more transmissible variants and mass vaccination.”

1.3 The authors write that they use an "individual-based" model but, unless I am mistaken, the individuals are not tracked. More precisely, the host population does not vary (there is no immunisation through natural immunity or vaccination). This is not an issue for the first time periods of course, but since the authors show results for mid-2021, although the cumulative incidence was not that high, it could have a disproportionate effect if one assumes that more connected individuals are more likely to be exposed (in which case they would also be more likely to be immune).

The reviewer is correct that individuals are not tracked in the model in the sense that the host population does not vary over time, i.e. there is no immunity through infection or vaccination. We described the model as “individual-based” since each index case has a fixed number of household contacts, each of whom can only be infected once, and non-household contacts sampled from the contact survey data, and we calculate the number of secondary infections for each of these index cases to determine the secondary infection distribution. However, we realise that using “individual-based model” in this way may be misleading, so have replaced it with “stochastic model”. As the reviewer points out, accounting for immunity through infection and vaccination in later time periods and the fact that more connected individuals are more likely to be exposed, and therefore immune, would have a significant impact on the estimated secondary infection distribution. This is why, as noted above, we only estimate it and present it in Figures 4 and 5 (old Figures 3 and 4), for 2020, since the assumption that the vast majority of individuals are still susceptible during this period is reasonable.

1.4 I was happy that the authors introduced confidence intervals for their plots on number of contacts in Figure 1. However, I am not sure that the binomial confidence intervals bring a lot. Another option, which we used with one of these contact datasets, could be to discretise the data (e.g. per week) and use these weekly values to generate a distribution of heterogeneity values. This would have the advantage to illustrate how variable the polls are from one week to the next.

We thank the reviewer for this suggestion, but feel that since our analysis of the secondary infection distribution over time uses the whole contact distribution for each time period it makes most sense to present the results in a simple way per time period, while still providing some information about the uncertainty in the estimates of the proportions reporting more than a certain number of daily contacts.

1.5 A large part of the focus of the manuscript is devoted to the implementation of self-testing but it was unclear to me how this was modelled exactly (there are only 4 lines in the main text and none in the Supplementary materials). Also, the link between testing and symptoms is not mentioned (I could only find a discussion regarding self-isolation and symptoms). Finally, I think it would be interesting to show whether the implementation of self-testing has a different effect in a model without heterogeneity compared to a model with heterogeneity (in a scenario that is not a lock-down of course). This would help better demonstrate the importance of the current formalism.

We agree that the description of the model of self-testing in the Methods was too brief as some of the relevant information was in the Results and have now expanded the information as follows:

“We determined the probability of testing positive on an LFT at a given viral load, p_{test}, by fitting a logistic regression model to data on LFT outcomes for isolates with different viral loads[8]. Detection was then modelled via a Bernoulli draw with this detection probability, i.e.

$$test \sim Bernoulli(p_{test})$$

$$logit(p_{test}) = \alpha_0 + \alpha_1 vl$$

where $\alpha_0 = -9.1$ (95% CI: $-13.3, 5.0$) and $\alpha_1 = 1.8$ (95% CI: $1.1, 2.6$) were the estimated coefficients from the logistic regression.

We then modelled index cases testing regularly with LFTs at different frequencies (every 1, 3, and 7 days) or only before events of a certain size (before meeting >10, >20, >50 others) to determine the comparative effectiveness of rapid testing to control transmission. We assumed that a certain proportion of individuals self-isolated at home upon first testing positive, such that their non-household contacts were decreased to zero after the date of the positive test, but their number of household contacts was unchanged. The remaining individuals were assumed to continue making contacts as normal. We varied the proportion of individuals who self-isolated upon testing positive from 0-100% to model varying uptake/adherence. We assumed timing of testing was not affected by symptom onset and intensity, as we wished to model regular and event-triggered testing regardless of symptoms. We considered three indicative timepoints before (BBC Pandemic) and during (1st lockdown (March-June) and the reopening of schools (September 2020)) the pandemic.”

We also agree that comparing the impact of self-testing in a model without heterogeneity to one with heterogeneity is informative, so have added a supporting figure (Figure S6) showing the impact of different testing frequencies on *R* and *k* for the model with and without overdispersion in contacts. We have commented on this in the Results on p20 lines 674-678:

“From the sensitivity analysis comparing the impact of regular testing with and without overdispersion in contacts (Figure S6), we can see that ignoring overdispersion in contacts reduces the estimated impact of testing in terms of reducing *R* and decreasing the proportion of superspreading events. This demonstrates the importance of accounting for contact overdispersion when estimating the impact of interventions on secondary infections.”

2) References

One of the reasons why I stopped modelling COVID (besides the lack of funding), is that the bibliography changes at a huge pace. Here, given the public health focus of the article, I was expecting to read about some of the recent developments in the field. Unfortunately, among the 32 references, only 3 are from 2023 and 2 from 2024. This would of course not be a problem, even for such a rapidly moving field as the COVID one, if it only interfered with the introduction and discussion. However, I think this also impacts the methods and results.

2.1 I find the data used for the virus load kinetics a bit suboptimal, especially since in the end the authors use distributions with 3 parameters. The data originates from an NBA cohort where most of the individual tested are in excellent physical shape. Since then, new data has been released that seems essential to address the question here, especially data on long infections (e.g. see the work from Ghafari et al. 2024 Nature https://doi.org/10.1038/s41586-024-07029-4).

We agree that the NBA cohort is not representative of the general population, however it remains one of the most densely sampled longitudinal viral load datasets available with near daily PCR measurements which critically capture the critical early stage of infection. Ghafari et al. as part of the Office for National Statistics COVID-19 Infection Survey sampled weekly then monthly with the majority having less than 3 data points. Additionally, persistent infections beyond 60 days made up 0.1-0.5% of all infections, with PCR derived viral loads ~100-fold lower than at symptom onset, substantially reducing their transmission potential. However, we have now included an additional sensitivity analysis doubling the standard deviation of all viral load parameters (Figs S4 and S5) to account for greater variation in viral load trajectories in the general population. This does not change our qualitative conclusion that contact heterogeneity remains the primary driver of heterogeneity in transmission. We have added discussion of the non-representativeness of the NBA cohort, the Ghafari et al. paper, and the sensitivity analysis in the Discussion in lines 840-857, p 25.

2.2 The link between SARS-CoV-2 viral load and contagiousness is tricky to calibrate. The authors lean on a 2021 study based on the probability of culturing the virus but, as for the infection duration, there is more robust work linking virus load and transmission (see e.g. Marc et al. 2021 eLife https://doi.org/10.7554/eLife.69302)

Thanks for highlighting this very interesting paper. We believe the two approaches are complementary: Marc et al. inferred the viral load–infectiousness relationship from observed transmission outcomes in the Marks et al. 2021 household cohort, whereas we use culture positivity from Pickering et al. as a direct virological proxy alongside empirical contact survey data. We both reach similar conclusions — Marc et al. found a substantial residual random effect (SD = 85%) after accounting for viral load, indicating viral load alone does not explain transmission heterogeneity, consistent with the original Marks et al. finding that contact type (household OR ~3) was independently associated with transmission alongside viral load (OR ~1.3 per log10 increment). We have added to the Discussion section (lines 777-779, p 23):

"Our approach of using culture positivity as a direct virological proxy for infectiousness is complementary to that of Marc et al., who inferred the viral-load–infectiousness relationship from observed transmission outcomes and similarly found that viral load alone does not fully explain transmission heterogeneity (residual random effect SD = 85%)."

2.3 I was wondering how the results from the UK app. showing that contact duration matters more than the exact distance could affect the empirical fitting of the data. In particular, people could have more contacts than they expect if distance is less of an issue (Ferretti et al. 2023 Nature https://doi.org/10.1038/s41586-023-06952-2). More generally, discussing the usefulness of a contact app. to estimate Rt, e.g. the study by Kendall et al. 2024 Science https://doi.org/10.1126/science.adm8103) seems relevant in this context.

We thank the reviewer for raising these two papers. The finding from Ferretti et al. that duration rather than distance drives transmission risk is consistent with CoMix defining contacts as face-to-face interactions with reported duration, and implies that the contacts most likely to be missed in a survey

(brief, low-duration encounters) are also those that contribute least to transmission. Kendall et al.'s result that before Omicron, contact rates explained more of the daily variability in R(t) than per-contact transmissibility (46% vs 30%) is consistent with our finding that contact heterogeneity is the primary driver of overdispersion; their app-derived contact rate also tracked CoMix closely.. Notably, Kendall et al. identify as a limitation that the app could estimate mean contact rates but not their dispersion, which we can estimate from CoMix data. We have added a mention of Ferretti et al. and Kendall et al. in the Discussion (lines 779-783, p23):

"Consistent with our findings, Kendall et al. found that contact rates explained more daily variability in *R* than per-contact transmissibility (46% vs. 30%) using NHS COVID-19 app data that tracked CoMix closely; Ferretti et al. similarly found that contact duration drove transmission risk more than proximity, supporting the use of duration-weighted contacts in our model."

2.4 Should the authors decide to keep their results from 2021, there is also some literature about how temporal variations in Rt heterogeneity could be explained by spatial structure and the depletion of susceptible hosts (Thomine et al. 2021 eLife https://doi.org/10.7554/eLife.71417). There could also be an effect of variations in generation time (e.g. Blanquart et al. 2021 eLife https://doi.org/10.7554/eLife.75791).

We only ever estimated transmission heterogeneity for 2020 prior to widespread variant emergence, vaccination, and significant depletion of susceptibles, so feel that it is beyond the scope of our analysis to discuss how these factors may cause temporal variations in Rt.

3) Fit with the journal

3.1 The manuscript was reviewed by PLoS Biology and probably sent directly to PLoS Computational Biology without any modification of the format. However, even when bearing this in mind, PLoS Comput Biol may not be the best fit. The main reason is that the insights of the work will clearly appeal to public health experts rather than to computational biologists. Even I feel poorly qualified to assess the public health impact of this work, e.g. compared to earlier results regarding the effect of self testing, and I think this should be done by a more specialised journal.

We appreciate the reviewer raising their concerns here. However, we feel that the paper is a good fit for PLoS Comp Bio. PLoS Comp Bio has published many papers on infectious disease modelling, and particularly COVID modelling, and this model fits within this ilk of papers that use mathematical and computational models to improve understanding of infectious disease transmission. PLoS Comp Bio also has a broad readership, including infectious disease modellers and public health experts. The manuscript was in fact considerably revised before submission to PLoS Comp Bio in response to the concerns raised by the reviewers at PLoS Bio, who recommended that it be transferred to PLoS Comp Bio.

3.2 Related to the previous point, the strength of this work comes from the fact that the authors sample from empirical distributions (although questions can be raised about the dataset used for the virus load and about the variability of the one use for the contact data). This comes at a cost in terms of generalisation. I am unsure these results could be extended to other infections. Furthermore, does the use of empirical distributions (especially for virus load) yield different result than other families of functions consistent with the infectious duration of respiratory viruses? Again, with in a public health-oriented journal, this focused aspect of the research would be less of an issue.

We agree that the use of empirical distributions necessarily ties the analysis to the specific datasets used. However, we believe the framework is generalisable. Provided densely sampled viral load trajectories are available for a given pathogen, the trajectory model can be fitted. The relationships between viral load, culture positivity, and test detection are assay-specific quantities that must be estimated from pathogen-specific data in any case. Contact survey data with duration information, which already exists for many settings, can be used to characterise the contact distribution. We use the Pickering et al. data precisely because it provides all of these quantities for the same assay and

isolates, preserving the internally consistent correlations required. For other respiratory viruses, the same framework could be applied provided analogous longitudinal viral load and culture/test data are available. We therefore do not view the empirical approach as a limitation as it avoids excessive ad hoc assumptions about viral kinetics. We have added a brief note to this effect in the Discussion (lines 922-926, p27):

“The modelling framework is generalisable: provided densely sampled viral load trajectories and pathogen-specific culture/test sensitivity data are available to characterise infectiousness, and a contact survey with duration information is available to characterise the contact distribution, the same approach can be applied to estimate heterogeneity in the secondary infection distribution for other respiratory pathogens. ”

3.3 Writing this piece for a less computational journal would also allow the authors to go into further details regarding the drivers of heterogeneity. I only glanced at the two articles they cite but neither of these seem to investigate these individuals with high number of contacts. However, they focus a lot on other criteria such as age. It would be interesting to see if these individuals with high number of contacts differ from the rest of the population because it would clearly impact the possibility to control the epidemic.

We agree that it would be interesting to look at whether high-contact individuals differ in terms of characteristics from the rest of the population, but we feel that this is beyond the scope of the current analysis, which focuses on the potential impact of rapid testing as a means to prevent superspreading.

Detailed comments

line 252-254: Since Figure 3 was simulated using the data shown in Figure 1, isn't it expected that they look alike?

What we intended to convey was that our model-derived R estimates, derived from contact rates, are broadly consistent with SPI-M consensus R estimates derived from observed infection data (Figure 4). We have updated that section to read:

“Our analysis shows that changes in the mean and overdispersion of daily numbers of contacts over time (Figure 2C) can be used to predict dynamic shifts in the mean and overdispersion of the reproduction number (Figure 4) . This motivates collecting contact data during epidemics, as contact overdispersion could provide a potential indicator of superspreading risk in real-time, along with the effective reproduction number. Changes in contact overdispersion would indicate when and where the population is at risk of superspreading events, and enable implementation of targeted, rather than blanket, restrictions on contact levels to limit outbreaks. However, ground-truth data on secondary infection distributions, e.g. from contact tracing studies, would first be required to validate estimates of the mean and overdispersion of the secondary infection distribution.”

line 407: It might be interesting to compare the negative binomial distribution to others (line 407).

We thank the reviewer for this suggestion. We compared the negative binomial (NB) to a zero-inflated negative binomial (ZINB) for three indicative time periods (Pre-pandemic, 1st lockdown, School reopening). The NB was found to be sufficient in all periods: the estimated zero-inflation parameter was effectively zero ($\pi \approx 0$), the ZINB provided no improvement in AIC ($\Delta AIC = +2.0$), and a likelihood ratio test gave $p > 0.99$. Full results are reported in new Table S2. We have added this comparison to the Methods (see p5, lines135-141).

Table 1 seems redundant with Figure 1.

We disagree that Table 1 is redundant with Figure 1 as it provides additional information on the dates

and sample sizes of each of the time periods shown in Figure 1. We would be happy to move this table to a supplementary file if the reviewer thinks this would be preferable.

[signed and dated]
- Samuel Alizon, Nov 1, 2025

Reviewer #2: # SUMMARY

This analysis aims to quantify the relative influence of variability in daily contacts versus viral shedding on heterogeneity in onward transmission for SARS-CoV-2 using early pandemic contact data from the UK. Overall, it is an interesting question and the overall approach comparing 4 main scenarios seems reasonable. Some methods details were not clear enough, and some conclusions are a bit strong given the analytic framework. However, my main concerns are how correlations in contact data and durations are implemented, which does not make best use of the available data to answer this question.

MAJOR ISSUES

1. **Contact correlations:** The methods note that household (HH) contacts were fixed after one random assignment, while non-HH contacts were sampled randomly each day. This makes sense to me for HH, but it's not clear from which distribution non-HH contacts were sampled. Did this sampling maintain correlations in a) individual-level contacts over time (e.g. some individuals consistently reporting more contacts than others), and b) durations of contacts (e.g. durations are likely shorter for individuals with many contacts).

1.a) I reviewed the results, methods, appendix, and github code, and I'm still unclear what's been done. From the code I think these correlations are ignored:
> https://github.com/bquilty25/superspreading_testing/blob/master/scripts/utils.R#L460

The reviewer is correct that previously we were ignoring these correlations and sampling HH contacts and non-HH contacts independently from the survey data for each time period. We have now updated this so that we are sampling HH contacts and non-HH contacts and durations for the same survey participant, to account for correlations between numbers of HH and non-HH contacts, in individual-level numbers of non-HH contacts over time, and between number of contacts and contact duration. We have described this in lines 190-207 on p7-8 under “Contacts and contact durations” in the Methods section (now moved before the Results).

1.b) Using the UK CoMix data, I estimated that at least half of the variance in non-HH contacts is essentially explained by individual-level effects (versus time effects or random variations) [code below]. While reported contacts in CoMix do not reflect subsequent days as in the current analysis, this correlation still suggests non-negligible individual-level effects.

Please see response to 1.a).

1.c) I expect that correct accounting of within-person correlation in contact daily non-HH contact numbers would drive even greater overdispersion in transmission due to contact heterogeneity, while accounting for duration correlations would have the opposite effect. It's not clear if these competing biases are of similar magnitude. Regardless, both correlations should be accounted for here, given the centrality of quantifying overdispersion to the research question, and it should be possible given the available data.

Yes, accounting for within-person correlation in daily non-HH contact numbers, and between HH and non-HH contact numbers, does drive greater overdispersion in transmission, and accounting for correlation between contact number and duration reduces it. These correlations have now been accounted for, but the effect of within-person correlation in daily numbers of non-HH contacts over time, and in numbers of HH contacts and non-HH contacts, is greater than that of correlation between contact number and duration. Our previous conclusion that contact heterogeneity contributes more to heterogeneity in numbers of secondary infections therefore still appears to stand.

1.d) A follow-on issue is that the current analysis ignores the influence of contact heterogeneity on *acquisition* risk. This is briefly mentioned in the limitations [324]. If individuals' contacts are correlated over time, we would not expect the distribution of contacts among infected individuals (at risk of transmission) to match the population-level contact distribution (estimated in surveys) -- at least prior to widespread immunity. Correct accounting of this aspect would likely increase R(t) substantially, though the implications for overdispersion aren't obvious to me. Again, it should be possible to examine this given the available data.

We agree that individuals acquiring infection during epidemic spread are likely to be biased toward those with higher contact rates, particularly if contact behaviour is persistent over time. However, incorporating this effect would require additional assumptions regarding how acquisition risk scales with contact number and network structure. While onward transmission from a known infectious individual can be modelled directly from observed contacts, acquisition risk depends on infection prevalence, assortative mixing, clustering, repeated contacts, and other higher-order network effects that are not identifiable from the contact survey data alone. In particular, weighting acquisition risk directly by contact number would implicitly assume approximately homogeneous mixing and independent exposure opportunities – assumptions we do not wish to impose without additional supporting data. We have added the following in the Discussion (lines 880-885 on p26) to note this:

"For instance, we would expect individuals that acquire infection to typically have higher contact rates than the average member of the population, since having more contacts increases acquisition risk, and therefore to infect more individuals. However, incorporating this effect would require additional assumptions about how acquisition risk scales with contact number and network structure that we do not have the data to support."

2. **Contact durations:** While I appreciate the authors' efforts to account for contact durations, I am concerned about the implementation: dur_{(N)HH,i,j,t} are "defined as the proportion of a 24-hour period spent at home or outside of the home respectively that the contact lasted". So, if someone spends 16/24 hours at home and 8/24 hours outside, and they report two 1-hour contacts: 1 at home and 1 outside, the household contact would then have "dur" = 1/16 while the outside contact would have "dur" 1/8, making outside contacts (in this case) "doubly infectious". More generally, the weight would be inversely proportional to the time spent in one or another context.

We apologise for the confusion here, which is due to poor wording of the definition of the contact duration. The contact durations are in fact defined as the proportion of a 24-hour period that the household/non-household contact lasted, so that there isn't the issue with inverse weighting of individuals' infectiousness with time spent at home/outside as the reviewer describes. We have now amended the wording to:

"Each household and non-household contact had a duration, $dur_{HH,i,j,t}$ and $dur_{NHH,i,j,t}$, defined as the proportion of a 24-hour period that the contact lasted (Figure S1)."

MEDIUM ISSUES

1. **Structure:** As with other similarly structured papers, the methods-last structure (suggested by the journal) feels inefficient and sometimes confusing. Various methods details are necessarily given in the results section, but these are inevitably repeated with some added detail in the final methods section, or with still more details sometimes in the appendix. I was never quite sure where to expect the complete details of any analysis step during first/second read. Some aspects aren't actually mentioned in the methods, such as pre-event testing.

We agree with the reviewer that the paper is better served by having the Methods before the Results and that the details of the modelling were spread across too many locations. We have therefore moved the Methods section before the Results and combined it with the information that was in the supplementary material (pp 4-10 lines 98-275), and moved relevant details on the methods that were in the Results to the Methods. We have also included further details on regular and pre-event testing

and the sensitivity analyses that we conduct under “Simulation of interventions” and “Contribution of contact and viral load heterogeneity to heterogeneity in secondary infections”.

2. **Overdispersion parameter:** It's quite counterintuitive that the chosen overdispersion parameter (k) decreases with variance. It's helpful this has been stated explicitly in a few places, but the reader must constantly be reminded of this counterintuitive relationship. Why not simply use the reciprocal \alpha = 1/k instead, as is sometimes done elsewhere? Or perhaps the coefficient of variation (SD/mean). It would be much easier to follow, throughout.

2.a) This would also help with the disappearing "all-homogeneous" case in Figure 4.

We acknowledge the reviewer's point, but feel that it is best to present the results in terms of k rather than 1/k or the coefficient of variation, even if it is less intuitive, for consistency and ease of comparison with other papers that look at superspreading, since the majority of papers use the parameterisation in terms of k.

3. **Falsifiability:** I am inclined to agree with previous reviewers that it would be difficult to draw different conclusions (e.g. that VL variation actually is drives super-spreading more than contact heterogeneity) based on the present analysis.

3.a) Perhaps if the (presumably) negative correlation between daily contact number and durations were captured, then this would allow VL heterogeneity to exert more influence in those contexts with super-spreading potential.

We have now captured negative correlation between daily contact number and duration (Figure S2) by sampling contact numbers and durations from the same survey participants. However, our results still show greater heterogeneity in secondary infections in a scenario with no variation in viral load and overdispersed contacts than in a scenario with Poisson contacts and variable viral load (Figure 5). This is even the case when we double the standard deviations of the viral load trajectory parameters to model much greater variation in peak viral load and shedding duration (Figure S5). Taken together, these results suggest that contact heterogeneity contributes more to heterogeneity in secondary infections than viral load heterogeneity for SARS-CoV-2.

MINOR ISSUES

- [55+] I appreciate the authors' effort to succinctly label the two competing hypotheses here as "wrong-person" versus "wrong-time". However, the word "wrong" feels ... wrong here, and possibly vaguely stigmatizing (drawing on HIV/mpox work). Perhaps something like "between-person (variability)" versus "within-person variability" could better capture the idea?

We agree that “wrong person” has unfortunate connotations. Hence we have relabel the competing hypotheses as “between-person infectiousness variability” and “between-person contact variability and within-person infectiousness variability”.

- [100] Figure 1a: overall, this is a great illustration of these trends. Can a sequential colourmap be used here to make the trend across contact numbers more intuitive?

Thank you for the suggestion - a sequential colour map has now been used.

- [112] Table 1: is "N" here the number of unique respondents or survey responses? I assumed the latter, but it may be worth clarifying in a table note - and likewise for the (%) columns.

N is the number of survey responses. We have added a footnote to clarify this as suggested.

- [142] I had to re-read this very long sentence several times to understand everything being said: "By mapping viral load to infectivity via a logistic function based on viral load and culture (representing

live, infectious virus) from Pickering et al. 8 (Figure 2B), calculating P(infectivity) by day (Figure 2C), then integrating under the infectivity curve, we can reproduce substantial heterogeneity in individual infectiousness as reported by Ke et al.6, with a >70-fold difference in individual-level infectivity between the 2.5% and 97.5% percentiles of the individual-level distribution (0.08 and 5.74, a.u, respectively) and a shape parameter of a fitted Gamma distribution of 1.42." Please consider breaking this up into shorter, clearer sentences. For example, is gamma distribution fitted to the density of relative infectivity over time? This is not clear, even after reviewing the corresponding methods text.

As suggested, we have broken this sentence up into shorter, clearer sentences:

"Mapping viral load to infectivity via a logistic function based on viral load and culture from Pickering et al. [8] (Figure 3B), calculating P(infectivity) by day (Figure 3C), then integrating under the infectivity curve to calculate total infectious virus shed, reproduces substantial heterogeneity in individual infectiousness as reported by Ke et al. We found a >70-fold difference in individual-level infectivity between the 2.5% and 97.5% percentiles of the distribution of the area under the infectivity curve (0.08 and 5.74 arbitrary units (AU), respectively). Fitting a Gamma distribution to this distribution gave a shape parameter of 1.42 (1.6 for Ke et al.)."

- [148] "When simulating whether or not individuals would infect a single contact on each day of their infection ..." I was confused reading this: have contact rates analyzed in the prior section now been incorporated? If so, for which time period?

No, contact rates from the previous section had not been included here; the plot was showing the distribution of the number of days each individual infected a single contact of the median contact duration. However, as the reviewer notes, this was confusing, as we were mixing information about potential infectiousness based on viral load with information on realised infections. We have therefore changed Fig. 3E to show the distribution of the number of days individuals are infectious (i.e. have the potential to infect others) based on the mapping of their viral load to relative infectivity.

- [165] I found the term "individual-based model" a bit misleading, as I was expecting a model that captured iterative acquisition/transmission chains, which is usually what "IBM" means in ID modelling. I recommend to use a different term, perhaps simply "stochastic model".

We agree that "individual-based model" is potentially misleading and have therefore changed this to "stochastic model" as suggested.

- [243] The statement "closely matching contemporaneous estimates" is a bit strong, given that all the R(t) estimates hover around 1.

We have reworded this to "are consistent with contemporaneous estimates".

- [248+] "dispersion in the reproduction number" is a bit strange to say, since the reproduction number is defined as the *mean* number of secondary cases per index case. Maybe "dispersion in onward transmission"?

We agree and have reworded this as "dispersion in the number of secondary infections" and in the following sentence as "overdispersion in the secondary infection distribution".

- [267] I do not follow the logic of: 'superspreading is more a case of "wrong place, wrong time" than "wrong person".'

This may have been poorly expressed, but the idea was that superspreading results more from being one of a large number of contacts of an infected individual (“wrong place”) who is at/near their most infectious (“wrong time”) than making contact with someone who is a lot more infectious than others (“wrong person”). We have now reworded this sentence as:

“This indicates that superspreading is more a result of between-person contact variability and within-person infectiousness variability than between-person infectiousness variability.”

- [351] "other respiratory viruses that exhibit heterogeneous transmission, including influenza, RSV, and adenoviruses" can a citation be given here?

Certainly. We have added references to the Leung 2021 Nature Reviews Microbiology paper on “Transmissibility and transmission of respiratory viruses”, the Lloyd-Smith et al. Nature 2005 superspreading paper, and the Goyal et al. eLife 2021 paper, all of which discuss heterogeneity in transmission of respiratory viruses.

- [440] Can the authors clarify the maximum number of days post-exposure that were considered for onward transmission? Relatedly, the actual process of iterating over each day for each individual is only implied when the Bernoulli sampling is described, but this iteration could be stated more plainly.

We have now explicitly stated that the Bernoulli sampling is iterated over each day of infection and clarified the maximum number of days post exposure that were considered for onward transmission in the Methods (lines 217-223, p8):

“This process was iterated over each day of the index case’s infection up to a maximum infection duration of 44 days (in the baseline analysis).”

and for the viral load heterogeneity sensitivity analysis in lines 245-248, p9:

“We reran this analysis with greater variation in viral load trajectories across individuals, by doubling the standard deviations of the distributions for the proliferation time, clearance time and peak Ct value, to test the sensitivity of the results to the degree of viral load heterogeneity. For this sensitivity analysis, we considered onward transmissions up to 88 days post exposure.”

- [github] The "utils.r" contains many steps of analysis code that I was surprised to find in a file called "utils"

We realise that the naming of this file as utils.R may not be that conventional but have decided to keep the model functions in this file for simplicity and to maintain the history of modifications of the functions for anyone that would like to examine it.

CODE

Here is code I used to estimate the variance explained by individual-level effects in CoMix UK:

```R
X = merge(read.csv('CoMix_uk_contact_common.csv'),
read.csv('CoMix_uk_participant_extra.csv'))
X = cbind(n=1,subset(X,!cnt_home)) # nhh contacts
A = aggregate(n~survey_round+panel_id,cbind(n=1,X),sum)
names(A) = c('t','id','n')
# complete dataset is too big: ah hoc bootstrap
S = do.call(rbind,parallel::mclapply(1:1000,function(i){
set.seed(i); ll = logLik
A.i = subset(A,id %in% sample(A$id,100,rep=0))
A.i[1:2] = lapply(A.i[1:2],as.factor)
m0 = glm(n~1, 'poisson',A.i)
```

```
mt = glm(n~t, 'poisson',A.i)
mi = glm(n~id, 'poisson',A.i)
mit = glm(n~id+t,'poisson',A.i)
S.i = data.frame(
d0=m0$dev,dt=mt$dev,di=mi$dev,dit=mit$dev,
l0=ll(m0),lt=ll(mt),li=ll(mi),lit=ll(mit))
},mc.cores=7))
# a few pseudo-R2 options ...
summary(1-S$dit/S$dt) # R^2_L (a) ~ 0.79
summary(1-S$di /S$d0) # R^2_L (b) ~ 0.68
summary(1-S$lit/S$lt) # R^2_CS (a) ~ 0.64
summary(1-S$li /S$l0) # R^2_CS (b) ~ 0.59
```

Reviewer #3: In the manuscript by Quilty et al., the authors combine previously published survey data and previously published within host virus data to model COVID-19 dynamics. Their particular focus is on measuring heterogeneity in transmission (i.e. superspreaders), and whether superspreading is more closely tied to contact rate or infectiousness. They conclude that contact rate, as opposed to infectiousness, is the key driver of heterogeneity. They go on to talk about testing rates and lockdowns and the influence these things had (or could have) on disease dynamics. Overall, I thought this was a very interesting manuscript, but I have numerous suggestions for improvement.

First, I think the authors should take some time to elaborate on a conceptual issue with these types of "which is more important A or B?" type studies. That being, if one variable is measured more accurately than the other, the more accurately estimated thing will appear more important even if it isn't. In this case, the hosts measure of "contact rate" is a direct measure of contact rate, whereas virus load within hosts is a proxy for infectiousness. There are sources of variation in infectiousness that may be strongly decoupled from within-host dynamics, which could greatly increase the variation in transmissibility attributable to infectiousness. These things might include innate factors (propensity to cough, antibody cross-reactivity) or behavioral factors distinct from contact related things (i.e. wiping nose vs. blowing, how close you stand to people, how loudly you talk). As the authors say in lines 422-425, Ke found heterogeneity in infectiousness that extended beyond the within host dynamics, and so the decision to leave it out potentially biases their results to find that contact rate is proportionally more important than it should be and infectiousness is proportionally less important.

We agree this is an important structural limitation: because contact heterogeneity is measured directly while infectiousness heterogeneity is captured only via viral load as a proxy, our analysis will structurally favour contact heterogeneity regardless of the true biology. We have acknowledged this explicitly in the Discussion (lines 863-872, p25), noting that factors decoupled from viral load dynamics are not captured and would, if substantial, increase the relative contribution of infectiousness heterogeneity.

We have addressed this in two ways. First, the logistic regression coefficients linking viral load to culture probability are now drawn per individual from the estimated covariance distribution (Methods, lines 172-178, p6), so two individuals with identical viral load trajectories still differ in their day-to-day probability of being infectious - capturing a portion of the extra-VL heterogeneity described by Ke et al. Second, we ran a sensitivity analysis doubling the standard deviations of all three viral load trajectory parameters (Figs S4 and S5). Even under inflated infectiousness heterogeneity, a scenario with overdispersed contacts and homogeneous viral load produced greater overdispersion in secondary infections than one with Poisson contacts and variable viral load. Thus we still conclude that the contribution of contact heterogeneity exceeds that of viral load heterogeneity.

Updated Discussion: "It was not possible to compare our within-pandemic estimates of k against published estimates for each time period, as such estimates are not available. The specific proportions of infectious individuals reported above depend on the fitted logistic dose-response curve between viral load and culture probability; alternative functional forms would yield different estimates. A structural asymmetry in our comparison is that contact heterogeneity is measured directly from survey data, whereas infectiousness heterogeneity is captured only via viral load as a proxy. Sources of between-individual variation in infectiousness that are decoupled from within-host viral dynamics -

such as variation in respiratory droplet production, speaking volume, or interpersonal distance preferences - are not captured, and if substantial would increase the true contribution of infectiousness heterogeneity relative to our estimates. We partially address this by drawing the logistic regression coefficients linking viral load to infectiousness for each individual from the estimated covariance distribution, and by examining robustness in a sensitivity analysis with twice the standard deviation in viral load trajectory parameters (Figure S5); however, these measures do not capture variation in transmission efficiency that is entirely independent of viral load. We also do not account for correlation of the timing of peak viral load and infectiousness with symptom onset and intensity, which may affect contact rates and numbers of secondary infections."

Another important point that the authors don't currently discuss is biases/limitations in their survey data that could lead to "fake" overdispersion. I am certainly not an expert with these types of data, but I know a bit about them. A challenge that I have seen with contact tracking data is that different people might report the same event differently. For example, two people go to dinner with each other. One might say that they had one contact, and the other might say that they had 50 contacts (i.e. number of patrons in the restaurant). Of course, in practice their effective number of contacts is the same, and somewhere between one and 50. It would be nice to hear a little more about this survey and how they did or did not control for this issue. Of course, there is no perfect solution and the impact of this should be discussed. Just using this example as an illustration, the authors' model would predict that there was drastic overdisperion, with one of the individuals being a superspreader and the other not even though the only difference here is in the reporting.

We agree and have added this explicitly as a limitation in the Discussion. We now note that while BBC Pandemic and CoMix define contacts as direct face-to-face interactions (which reduces ambiguity), there may still be between-individual variation in how participants interpret and report contacts, which can inflate apparent contact overdispersion. We also make clear that this could bias estimated k downward (toward more apparent overdispersion), so results should be interpreted with this uncertainty in mind.

Updated text (line 834, p24): "Additionally, variation between participants in how direct contacts are interpreted and reported could contribute to artificial overdispersion in the contact distribution and hence in our estimates of secondary infection overdispersion."

There were also a few places (particularly the first two paragraphs of the discussion) where the conclusions of the models seemed overstated. On lines 242-243 the authors state that they were able to reconstruct the secondary infection distributions from first principles. In reality, they showed that they were able to reconstruct the "mean" number the secondary infections, which is seems like a very different statement to me. Unless I missed it, Fig 3 shows they are doing a reasonable job on the mean, but does not include real data on overdispersion. Also, on lines 252-255, "Our analysis shows that dynamic shifts in the mean and overdispersion of the reproductive number closely track the changes in the mean and overdispersion of daily number of contacts," but I think this is an overstatement of their results. Really what they are doing here is showing that the model predicted overdispersion matches the overdispersion in their contact rate dataset (which it should since this is essentially a parameter in the model). The next sentence is equally an overstatement of the results, "… contact overdispersion can be reliably measured in contact surveys and is predictive of superspreading." As far as I can tell, saying it is "reliable" or "predictive" is not justified since there is no ground-truth data used in this manuscript to compare against. Likewise, the authors say (lines 263-264) that ">50% estimated to have viral loads capable of infecting many others on at least one day of their infection". As far as I can tell, this statement is highly dependent on a model assumption that infectiousness can be equated with the probability of culturing virus. Had they made a different assumption here, that 50% number could presumably be drastically different.

On estimating overdispersion: We would note that while we cannot validate our estimates of k in each of our time periods as we can for the mean reproduction number, we do compare our estimates for the baseline k (0.48) to other estimates such as that of Lau et al. (0.32 to 0.49 in counties across Georgia, USA) and Kremer et al. (k = 0.43 from contact tracing data in Hong Kong). Our estimate however is higher than that estimated by Endo et al. (0.1, 95% CI: 0.05 – 0.2). We have made this more clear in the Discussion section.

Lines 718-720, p22: “Our estimates of the degree of overdispersion in the secondary infection distribution are similar to those reported by other authors; estimates from contact tracing studies are mostly between 0.3 and 0.6, though lower estimates (k ≈ 0.1) have been reported from outbreak cluster size data.”

Lines 859-861, p25: “Independent empirical validation of the overdispersion parameter k across all time periods is not available here, and our within-pandemic estimates of k should therefore be interpreted as model-derived.”

On changes in the reproduction number tracking changes in contacts: (as in our response to Reviewer 1’s comment, repeated here for clarity) This section was poorly worded. What we intended to convey was that our model-derived R estimates, derived from contact rates, are broadly consistent with SPI-M consensus R estimates derived from observed infection data (Figure 4). We have updated that section to read:

Discussion (lines 722-730, p22): “Our analysis shows that changes in the mean and overdispersion of daily numbers of contacts over time (Figure 2C) can be used to predict dynamic shifts in the mean and overdispersion of the reproduction number (Figure 4) . This motivates collecting contact data during epidemics, as contact overdispersion could provide a potential indicator of superspreading risk in real-time, along with the effective reproduction number. Changes in contact overdispersion would indicate when and where the population is at risk of superspreading events, and enable implementation of targeted, rather than blanket, restrictions on contact levels to limit outbreaks. However, ground-truth data on secondary infection distributions, e.g. from contact tracing studies, would first be required to validate estimates of the mean and overdispersion of the secondary infection distribution.”

On >50% with viral loads capable of infecting many others: We agree that the reported proportions depend on our chain from viral load to infectiousness, and have added appropriate caveats. However, we do not consider this chain to rest on an assumption: each link is empirically grounded. We have therefore moved the justification of this in the Methods (Infectiousness section), and added a caveat about the dose-response curve in the Limitations: (i) the logistic relationship between viral load and culture positivity is fitted directly from data in Pickering et al.; (ii) culture positivity is the best available correlate of the infectious period, outperforming both PCR and symptom monitoring (Drain et al.); and (iii) lateral flow test positivity closely tracks viable virus in controlled human challenge studies (Killingley et al.), completing the link between viral load and test detectability. The resulting distribution of individual infectiousness (gamma shape ~1.42) is consistent with an independent estimate of ~1.6 from Ke et al.. We have also reworded the manuscript to report the proportion at two thresholds (~68% exceed a peak culture probability of 0.6, ~52% exceed 0.8), showing the conclusion is robust across a range of thresholds.

Updated Methods (Infectiousness) text (lines 178-182, p6): "We use culture positivity as our proxy for infectiousness, supported by an empirically grounded chain from viral load to infectiousness to test detection: (i) viral load determines the probability of culturing viable virus, with the logistic relationship fitted directly from data in Pickering et al. ; (ii) culture positivity is the best available correlate of the infectious period, outperforming both PCR and symptom monitoring (Drain et al.); and (iii) LFT positivity closely tracks viable virus in controlled human challenge studies (Killingley et al.)"

Updated Results text (line 345-249, p15): "We found a >70-fold difference in individual-level infectivity between the 2.5% and 97.5% percentiles of the distribution of the area under the infectivity curve (0.08 and 5.74 arbitrary units (AU), respectively). Fitting a Gamma distribution to this distribution gave a shape parameter of 1.42 (1.6 for Ke et al.). A substantial proportion (19.1%) of individuals were estimated to be infectious for zero days, but the remainder were infectious for at least one day (Figure 3)."

Updated Limitations text (line 862, p25): "The specific proportions of infectious individuals reported above depend on the fitted logistic dose-response curve between viral load and culture probability; alternative functional forms would yield different estimates."

Smaller points:

- As said earlier, sometimes the authors had a propensity to overstate their results, but in other places, they sort of seem to miss what I would consider to be the most interesting bits. Lines 28-30, the authors talk about testing every day to reduce the reproductive number. This feels sort of obvious and selling short the punch of the paper. I think it is because of the "test every 3 days bit". I feel like it would be a lot more interesting to reframe it as, "having everyone in the population test every x days would be equivalent to having people test only before events with minimum size y". To me, that sounds punchier, and it also highlights the main advance of the paper, while providing a clear use-case for this type of modeling approach.

We agree this is a nice framing and have updated the Abstract (p2 lines 28-32) to emphasise this equivalence between the two different (and more actionable) testing approaches.

- I realize that the format for this journal is to have the methods at the end, but around line 165, I felt like I needed to know a little bit about what the model looked like before I could understand/believe the text. It might be possible to put in a short (e.g. 1-3 sentence) description here.

We have now moved the Methods up before the Results section.

- Lines 216-219. This result seems confounded with the functional relationship between load and test detection rate. It would be cleaner to potentially remove the assumption of a correlation here and instead say about how the effect would be even stronger if there was a relationship between within host load and test detection rate.

The relationship between viral load and LFT detection is not an assumption but is fitted to empirical data from Pickering et al., who measured both culture results and LFT results on the same specimens. Removing this relationship would itself be an assumption - that LFT sensitivity is independent of viral load - which the data contradict. We have therefore stuck with using the relationship between viral load and probability of LFT positivity found by Pickering et al.

- Line 249-250. The authors talk about the degree of overdispersion as being between 0.3 and 0.6 but do not provide units for interpretation. Is this meant to be “k”? Also, referring to “k” as overdispersion (such as in Fig 4) is confusing because overdispersion is greatest when k is small. Better would be to say that k is an inverse measure of overdispersion. Most accurately though, I would say that overdispersion is 1/sqrt(k), i.e. the coefficient of variation.

We have updated axis labels and text to make clear that k is an inverse measure of overdispersion, while retaining k for consistency with the literature on superspreading (lines 89-90 on p3).

- Line 269. I’m either confused or there is a typo. The authors say that interventions can be targeted at individuals during their period of high infectivity by testing their contacts, but this would presumably be a delayed response that would be useless because you would only know that someone was a superspreader after they left their highly infectious period.

This was confusing wording - we meant that frequent testing of contacts of an index case could prevent transmission from those contacts (as in the cited paper), not further transmission from the index case.

Discussion (lines 766-769 on p 23): “Hence if reductions in contact rates can be targeted specifically at individuals ahead or during their window of high infectivity (e.g., by encouraging contacts of cases to undergo regular self-testing and self-isolation upon a positive rapid test result)...”

- Figure 6, I don’t understand why one contact of Individual C seems to become infected and then revert back to non-infected. Maybe I am misinterpreting this figure?

The lighter grey dots indicating out of household contacts are sampled independently and are replaced each day, whereas the household contacts are sampled once and are not replaced. So that out of household contact was infected but did not come into contact with that individual again.

- Lines 452 and 453, the authors use the abbreviation LFTs here, which doesn't seem necessary since they made it through 95+% of the manuscript without needing it. I found myself having think pretty hard to figure out what they meant by it.

We have now added more detail to the methods on the simulation of interventions and how lateral flow test positivity is modelled (lines 249-273 on p10), so use "lateral flow test" many more times throughout the manuscript than before. We have therefore defined the acronym "LFT" at its first use in the Introduction (lines 82-83 on p3) and use it throughout the rest of the manuscript.
