# Supplementary material for: Disentangling the drivers of heterogeneity in SARS-CoV-2 transmission from data on viral load and daily contact rates
Source: PLoS Comput Biol. 2026 Aug 28;22(8):e1014715. doi: 10.1371/journal.pcbi.1014715 (PMC13552949; doi:10.1371/journal.pcbi.1014715)
Supplement: Supplementary file 11 [file pcbi.1014715.s014.docx]

**Additional Editor Comments:**

Thank you for your detailed response and revision. The reviewers are in agreement that the message and methods are much clearer, and the additional sensitivity analyses increase confidence in the robustness of your results. They have identified a small number of minor issues which should, I think, be straightforward to address, and made several suggestions regarding scope, model choices, and uncertainty, which I encourage you to consider.

We thank the reviewers for their careful re-reading of the manuscript and further comments and suggestions. We have addressed all of these as detailed below.

**Reviewers' comments:**

All line numbers below refer to the Word version of the manuscript with track changes “Manuscript_track_changes.docx” viewed in the desktop version of Word with “Show Revisions in Balloons” under “Mark-up Options”

Reviewer's Responses to Questions

**Comments to the Authors:
Please note here if the review is uploaded as an attachment.**

Reviewer #1: I thank the authors for considering my feedbacks. I now find that the key message (heterogeneity in contacts matter more than that in virus load regarding epidemic spread) is much clearer. However, I still have a few issues.

1) Clarifying the scope. From the title, the authors insist on their use of "data on viral load and daily contacts". However, for the virus load at least, what the really reuse are estimates. This could be clarified in the abstract and in the main text. The fact that this data is already published could also be stressed.

We have clarified the scope and the fact that the data is published as requested. Specifically, we have made the following changes:

- Abstract: “viral load and contact survey data” -> “published viral load estimates and contact survey data”
- Author Summary: “with data on viral load trajectories and infectiousness” -> “with published estimates of viral load trajectories and infectiousness”
- Introduction: “derived from viral load trajectories and infectivity” -> “derived from published data on viral load trajectories and infectivity”

2) The sociodemographic characteristics of the data used is still not presented in details. This is I think a major issue because these might explain some differences, e.g. on the pre- and the first post-pandemic period, if the participants to the BBC experiment are wealthiers or younger.

We have made several changes to present and discuss the sociodemographic characteristics of the general survey participants and how they differ for participants with a high number of contacts. We have added Fig. S3 showing the age distribution of the participants in the BBC Pandemic and CoMix contact surveys compared with the 2020 age distribution of the UK, and discussed it at the start of the Results section:

“The age distribution of all participants in the BBC Pandemic survey and of the participants in each of the nine periods of the CoMix survey is shown in Figure S3. Relative to the mid-2020 age distribution of the UK population, children and older adults (70+ years) are under-represented in the BBC Pandemic data. The age distribution of CoMix participants is representative of the general population (with slight under-representation of children ≤4 years and over-representation of adults aged 50-69 years) and remains fairly stable across the different time periods.”

We have also added Fig. S6 to show total, household, and non-household contacts by age and gender for the nine time periods of the CoMix data, and discussed it at the end of the “Changes in the distribution of social contacts” subsection of the Results (lines 741-813, pp 12-13):

“Figure S6, in which contacts are stratified by age as well household/out-of-household, shows that both household and out-of-household contacts in children (<18 years) remained higher than those in adults throughout 2020 and early 2021, with significant increases in out-of-household contacts of children during periods when schools were open. Contact levels were similar for males and females across all time periods.”

One of the issues with presenting more detailed information on the participants is that only very limited data on their sociodemographic characteristics is publicly available. We have therefore only presented data on their age and gender.

However, we have added further information on the sociodemographic characteristics of the survey participants and individuals with high numbers of contacts from other studies in the Discussion, and discussed the potential implications of this information for control:

“In their analysis of the CoMix data, Gimma et al [Gimma 2021] found that school-aged children consistently had higher numbers of contacts across both lockdowns and periods of relaxed restrictions than working-age adults, who in turn had higher numbers of contacts than older adults (60+ years). Contact levels also varied with employment status, with part-time-employed participants having more contacts than full-time or self-employed participants. Another analysis of the CoMix data that focused on individuals with high numbers of contacts [Chapman et al 2021] identified that most reports of high numbers of contacts came from work and school settings and tended to be reported by participants in certain professions (teaching, retail, administration, construction, medicine, social work, hospitality). Some of this work is considered essential, and keeping schools open where possible is a high priority, so opportunities for reducing these contacts may be limited. This highlights the potential value of regular rapid testing as a way of preventing superspreading even when some individuals have many contacts.”

Further discussion of the limitations of the BBC Pandemic contact data due to potential non-representativeness in the participants surveyed has also been added (lines 1486-1492, p22):

“Another limitation of the BBC Pandemic contact data is that working-age adults were overrepresented among the participants, while children under 13 years were excluded (Figure S3). It is possible that this may have led to some bias in the reported contact distributions. However, the similarity of contact patterns observed in the BBC Pandemic and UK POLYMOD datasets [Klepac et al 2020] suggests that the BBC Pandemic data is broadly representative of pre-pandemic contact patterns in the whole UK population.”

3) The authors decided to resimulate the individual kinetics data from the estimates found by Kissler et al. whereas for the contact data they use individual trajectories. If taking the individual Ct trajectories is not feasible because of the truncations, why not take the individual posteriors?

We decided to resimulate trajectories from the reported population-level parameter estimates to capture the population-level kinetics while maintaining a computationally tractable simulation framework. We acknowledge that propagating the posterior distributions of the individual-level parameters from Kissler et al. would more fully account for the posterior uncertainty from the original inference procedure. However, doing so would require multiple orders of magnitude more simulations. We expect the impact on our main conclusions would be limited because our analysis is driven primarily by population-level variation rather than uncertainty in individual parameter estimates, and uncertainty we have not accounted for should to some extent be captured under the sensitivity analysis in which we double the standard deviations of the posterior distributions of the viral load trajectory parameters.

4) Several references are off (they all point to Ref. 8 instead of the correct one). Furthermore, several figures (e.g. Figs 2C, 3D, or 6) are missing their confidence interval (or the caption is missing a description of what the shaded area exactly stands for).

Thank you. The references have been corrected. We do not present CIs on the plots in Figs 2C or 6. The shading under the curve in Fig. 3D was just a stylistic visualisation choice, but we agree that it is potentially confusing so have now removed it.

5) In the discussion, it could be worth extending the scope since this question of the importance of infectiousness vs. number of contacts can apply to any infectious disease.

We have extended the scope in the Discussion by expanding the section discussing the relevance of our work (lines 1626-1641, p24):

“... while our analysis is restricted to 2020, our findings are still relevant in the current era of endemic COVID-19 and to other pathogens that exhibit heterogeneous transmission, including SARS, Ebola, measles, influenza, RSV, and adenoviruses [Goyal et al. 2021; Leung et al. 2021; Lloyd-Smith et al. 2005]. Moreover, the question of the relative contribution of variation in numbers of contacts and variation in infectiousness to heterogeneity in transmission is a universal one for infectious diseases. By demonstrating that superspreading potential is closely linked to the overdispersion of social contacts and the timing of peak infectiousness, we have shown the importance of monitoring not only the mean effective reproduction number but also its variability. … The modelling framework is generalisable: provided densely sampled pathogen load trajectories and pathogen-specific culture or test sensitivity data are available to characterise infectiousness, and a contact survey with duration information is available to characterise the contact distribution, the same approach can be applied to estimate heterogeneity in the secondary infection distribution for other pathogens.”

Detailed comments

l.72-73: this is an assumption and should be presented as such.

We have changed “can be characterised as negative binomial” to “can be assumed to be” to avoid any ambiguity about this being an assumption.

l.88: Are kids allows to use their apps at school in the UK?

Yes, at the time they were allowed to. As explained in our response to point 2 above, participants in the BBC Pandemic survey were restricted to those aged 13 and over. We have now stated this at the end of the “BBC Pandemic” subsection to hopefully avoid any confusion:

“Children under 13 years of age were excluded from participating for ethical reasons,”

l.125: please remind the reader the exact dates.

The exact dates have been added.

l.133, 146c: wrong references

Thank you. These have been corrected.

l.140: This is data from synthetic T7 RNA transcripts. Is it the most appropriate?

Although the standard curve reported by Kissler et al. (2021) was generated using synthetic T7 RNA transcripts, this is a standard approach for establishing an absolute relationship between Ct values and RNA copy number because the transcript concentration can be accurately controlled. While synthetic transcripts may not fully capture all sources of variability present in clinical specimens, they provide a reproducible and well-characterized reference for converting Ct values to approximate genome equivalents. Since our objective is to obtain a consistent estimate of viral RNA abundance that can then be converted into a probability of culturing live virus, rather than an exact measure of infectious virus or total viral genomes in clinical samples, the Kissler et al. calibration is an appropriate choice. We acknowledge, however, that the resulting genome-equivalent estimates should be interpreted as approximate and dependent on the assumptions underlying the original standard curve.

l.195: How is beta set?

This is explained in lines 472-476 on p7, where we state

“We calibrated $\beta$ such that the basic reproduction number $R_{0}$, the reproduction number for the pre-pandemic time period, was 2.5 (based on estimates from the literature[^14,16,17^](https://www.zotero.org/google-docs/?SJQclC)) for the baseline analysis and all sensitivity analyses varying heterogeneity in contact rates and viral load. We obtained values of $\beta$ between 1.1 and 4.7.”

We feel that it makes more sense to explain how beta was fit after giving the definition of the reproduction number as the fitting is based on $R_{0}$.

l.322-323: the infectiouness duration in this model seems very low compared to the serial intervial estimates.

The median duration of infectiousness (not infection) – defined as having high enough viral load to culture virus – of 2 days is indeed shorter than the generation time/serial interval estimates for SARS-CoV-2 of 5-6 days. However, this is not surprising as the duration of 2 days we report does not start from infection; it refers to the index case’s period of peak infectiousness around when their viral load peaks. Based on the parameters for time from exposure to peak Ct from Kissler et al., we would expect the peak Ct (and therefore viral load) to typically occur 4-5 days after exposure, as the median viral load curve in Fig. 3A shows, which is in line with reported generation time estimates. We also note that 2 days is the *median* duration of infectiousness and that the 95% prediction interval ranges from 0 to 6 days, such that some individuals remain infectious for much longer.

We have added “around peak viral load” after “median duration of infectiousness” in line 622 on p15 to try to avoid confusion.

l.388: Perhaps also show the results with the doubled variance on this figure?

To avoid the figure becoming too cluttered and difficult to read, and as the results with the doubled variance are part of a sensitivity analysis, we prefer to keep the results in the separate supplementary figure Fig. S5.

[signed and dated]
- Samuel Alizon, 19 May 2026

Reviewer #2: Overall I am satisfied with the additional changes, in particular: the methods are much clearer now, and the added sensitivity analyses help ensure the headline finding is robust --- that contact heterogeneity likely plays a larger role than viral shedding heterogeneity in overdispersion.

A few additional minor points, which I leave to the editors / authors to decide if they are necessary to address in another round of revisions.

+ The methods section [108] "analysis of social contact data" and corresponding results section [257] "changes in the distribution of social contacts with changes in the intensity of restrictions" are not really introduced as an objective -- essentially a descriptive analysis of the data. While these are useful for the paper, they currently feel unexpected when reading on first pass based on the current introduction.

We thank the reviewer for noting this. We have now added the following sentence in the Introduction in lines 154-156 on p2 to make it clear that we first conduct a descriptive analysis of the data:

“As overdispersion in the social contact data has not previously been analysed or reported, we first perform a descriptive analysis of the social contact data.”

+ [223] It seems like we are missing a brief description about how the homogeneous viral load trajectory case is defined - presumably applying the mean parameters to everybody...

We had written here that:

“In the ‘homogeneous’ cases, … all individuals had the median viral load trajectory”

but we have now added

“(i.e. the trajectory with the median proliferation and clearance durations and median peak Ct value)”

after “median viral load trajectory” to clarify what we mean by this.

+ [225] It would be better to use the word "sensitivity analysis" in the first sentence of this paragraph, so it's clear we are deviating from the main analysis.

As suggested, we have changed the start of this paragraph to read “We performed a sensitivity analysis…”.

+ [600] I appreciate the added discussion of heterogeneous acquisition risk here. I might even add that, if anything, heterogeneity in acquisition risk would likely further amplify the relative importance of contact heterogeneity (versus heterogeneity in viral load trajectories), even under homogeneous mixing, and even more under homophilic mixing. Also, I am not really satisfied with the justification in the letter [R2.1.d] to avoid weighting acquisition risk by contact rates because it requires "additional assumptions" or data, since the current implementation makes arguably a stronger assumption implicitly, also without evidence: that acquisition risk is independent of contact rate. Nevertheless, leaving this as a limitation is acceptable to me.

We thank the reviewer for being willing to accept leaving this as a limitation of the present work as described in the Discussion.

+ [Figure 5] It's a hard to see the dotted vs solid line in the legend/guide, and the CI are hard to distinguish in the top panel. Why not use 3 distinct colours instead?

We have changed the figure to use three distinct colours for the different scenarios.

Reviewer #3: I am mostly happy with the revisions. One slightly bigger point and one smaller point remain.

The bigger point: The authors really want to say that a large fraction of individuals were infectious at some point in their infections, but I don't think they can justify this statement. The statement is built on this line: "culture positivity is the best available correlate of the infectious period, outperforming both PCR and symptom monitoring (Drain et al.);", but I looked into that manuscript and the statement is not justified by the publication. Drain et al. just assume that hosts are infectious when cultures are positive -- this is not based on any data in their manuscript. It also isn't biologically justifiable given that culture positivity (they aren't measuring TCID50, just yes/no) will depend on how much sample is used on each plate. As best I can tell, Drain et al. literally replace the term "culture positivity" with "infectiousness" and then look for other correlates of "infectiousness". This means that the conclusions in the present manuscript about the fraction of individuals infectious at various points in time are based on the faulty premise that culture positivity equates to infectiousness. Growth in culture is probably related to infectiousness, but the threshold for where something should be called infectious doesn't seem to be known (or at least, not from Drain et al.). This should at minimum be discussed as a limitation of the study. I get that the authors have done some sensitivity analyses, and so this doesn't seem like a fatal flaw, but I would encourage the authors to acknowledge the uncertainty.

We agree with the reviewer that our previous wording was too strong and not justifiable from the cited papers. We have therefore revised the relevant section in the Methods (lines 357-364 on p5) to:

“We use viral culture positivity as our proxy for infectiousness, because it indicates the presence of replication-competent virus and is therefore believed to provide a better indicator of transmission potential than Ct values from PCR measurements (Drain et al. 2023). It also allows us to make use of empirical relationships linking viral load, viral culture positivity, and test detection: (i) viral load determines the probability of culturing viable virus, with the logistic relationship fitted directly from data in Pickering et al. (Pickering et al. 2021) (Figure 3B); and (ii) LFT positivity closely tracks viable virus in controlled human challenge studies (Killingley et al. 2022).”

We had mentioned our assumption that the probability of shedding infectious virus is equal to the probability of culturing virus as a limitation in lines 1525-1528 in the Discussion, but have now expanded this and added a reference to be more explicit:

“We assume that the probability of shedding infectious virus is equal to the probability of culturing virus, but the exact relationship between viral culture positivity and infectiousness is not known (Puhach et al. 2023), so our results must be interpreted with this limitation in mind.”

Smaller point: The authors say in a few places (most notably the abstract and second discussion paragraph) that you can "predict" superspreading with contact rate data, but this is a strange choice of words, because (as the authors argue) the superspreading event is the heterogeneity in the contact rate data. And you can't predict something that has already happened.

We thank the reviewer for this observation. We were using the word “predict” in the sense of predicting a quantity using a model, not forecasting a quantity into the future, but realise that this was unclear. We have changed “predict” in the Abstract and Discussion to “estimate”, and in the Author Summary to “monitor”.
