# Supplementary material for: Disentangling the drivers of heterogeneity in SARS-CoV-2 transmission from data on viral load and daily contact rates
Source: PLoS Comput Biol. 2026 Aug 28;22(8):e1014715. doi: 10.1371/journal.pcbi.1014715 (PMC13552949; doi:10.1371/journal.pcbi.1014715)
Supplement: Peer review history

# Peer review: Decision Letter 0

**DOI:** 10.1371/journal.pcbi.1014715.r001

**Authors:** Nicholas Geard, Benjamin Althouse

19 Jan 2026

Disentangling the drivers of heterogeneity in SARS-CoV-2 transmission from data on viral load and daily contact rates

PLOS Computational Biology

Dear Dr. Chapman,

Thank you for submitting your manuscript to PLOS Computational Biology. After careful consideration, we feel that it has merit but does not fully meet PLOS Computational Biology's publication criteria as it currently stands. Therefore, we invite you to submit a revised version of the manuscript that addresses the points raised during the review process.

Please submit your revised manuscript by Mar 21 2026 11:59PM. If you will need more time than this to complete your revisions, please reply to this message or contact the journal office at ploscompbiol@plos.org. When you're ready to submit your revision, log on to https://www.editorialmanager.com/pcompbiol/ and select the 'Submissions Needing Revision' folder to locate your manuscript file.

Please include the following items when submitting your revised manuscript:

* A letter that responds to each point raised by the editor and reviewer(s). You should upload this letter as a separate file labeled 'Response to Reviewers'. This file does not need to include responses to formatting updates and technical items listed in the 'Journal Requirements' section below.

* A marked-up copy of your manuscript that highlights changes made to the original version. You should upload this as a separate file labeled 'Revised Manuscript with Track Changes'.

* An unmarked version of your revised paper without tracked changes. You should upload this as a separate file labeled 'Manuscript'.

If you would like to make changes to your financial disclosure, competing interests statement, or data availability statement, please make these updates within the submission form at the time of resubmission. Guidelines for resubmitting your figure files are available below the reviewer comments at the end of this letter

We look forward to receiving your revised manuscript.

Kind regards,

Nicholas Geard

Academic Editor

PLOS Computational Biology

Benjamin Althouse

Section Editor

PLOS Computational Biology

**Additional Editor Comments:**

The reviewers feel that your question and approach are interesting but raise several points that should be addressed for your manuscript to meet the requirements of publication in PLOS Computational Biology.

The key points raised include:

1. Justifying or acknowledging design choices regarding heterogeneities in duration of infection (R1), and number and duration of contacts (R2), and potential biases/limitations of data sources (for both viral kinetics and contacts) (R1; R3)

2. Clarity of methods (R1; R2; R3) – see note on manuscript structure below

3. Variation in epidemiological parameters over duration of analysis (ie, emergence of new variants and increasing population exposure) (R1)

4. Overstating conclusions (R2; R3)

5. R3 raises a pertinent point re the design of studies seeking to assess the importance of different drivers of an observed phenomenon, that warrants discussion

Note re manuscript structure: two reviewers commented specifically on the structure of the manuscript, and the infelicity of having Methods appear after Results for some types of study. Note that while suggested the default organization in the submission guidelines place Methods after Results, this is not a strict requirement. The submission guidelines also state: “To provide flexibility, however, authors are also able to include the Materials and Methods section before the Results section or before the Discussion section.” This may be appropriate here.

**Journal Requirements:**

1) Please ensure that the CRediT author contributions listed for every co-author are completed accurately and in full.

At this stage, the following Authors/Authors require contributions: Billy J Quilty, Lloyd A. C. Chapman, James D Munday, Kerry L M Wong, Amy Gimma, Suzanne Pickering, Stuart J D Neil, Rui Pedro Galão, W. John Edmunds, Chris I Jarvis, and Adam J Kucharski. Please ensure that the full contributions of each author are acknowledged in the "Add/Edit/Remove Authors" section of our submission form.

The list of CRediT author contributions may be found here: https://journals.plos.org/ploscompbiol/s/authorship#loc-author-contributions

2) We ask that a manuscript source file is provided at Revision. Please upload your manuscript file as a .doc, .docx, .rtf or .tex. If you are providing a .tex file, please upload it under the item type u2018LaTeX Source Fileu2019 and leave your .pdf version as the item type u2018Manuscriptu2019.

3) Please upload all main figures as separate Figure files in .tif or .eps format. For more information about how to convert and format your figure files please see our guidelines:

https://journals.plos.org/ploscompbiol/s/figures

4) We have noticed that you have uploaded Supporting Information files, but you have not included a list of legends. Please add a full list of legends for your Supporting Information files after the references list.

**Reviewers' comments:**

Reviewer's Responses to Questions

**Comments to the Authors:**

**Please note here if the review is uploaded as an attachment.**

Reviewer #1: Summary

The authors study the drivers of superspreading events in the case of SARS-CoV-2 (i.e. the fact that a minority of individuals causes the majority of the infections). More precisely, they with to determine whether these are caused by variations in virus load among individuals or whether they originate from behavioural variations. For this, they lean on existing databases regarding within-host temporal variations in virus load and temporal variations in the number of reported contacts. They use existing studies to perform numerical simulations with a simplified epidemiological model, e.g. without epidemiological feedbacks. The complement this model to explore counterfactual scenarios where later-flow testing can prevent transmission from individuals with high virus load. They interpret their results in the context of the literature, while highlighting potential limitations of their approach.

Review

I have three major concerns about the manuscript. First, although the model is designed to be simple, I find some of its assumptions overly simplistic. Second, the literature does not seem to be up-to-date for such rapidly moving field (there are few references from 2023 and hardly any from 2024). Third, I am unsure that the manuscript's strengths (public health insights) align with our journal's scope (mathematical and computational biology).

1) The model

The space devoted to the model is limited, even when accounting for the Supplementary Materials, but the modelling appears to be simple. In essence, the authors assume all the infections are independent (there is no depletion of susceptible hosts) and, for each infection initiated at time t, draw a number of household contacts and a number of non-household contacts (both of which depend on t) using empirical data. Every day of infection, they then perform a Bernoulli trial to determine if any of the contacts were infected or not.

1.1 My main critique about the model is that the authors tend to force the heterogeneity in virus kinetics to play in terms of the magnitude of the peak virus load. Another possibility, which I think would increase the window of transmission, would be to investigate the duration of the infection. As shown in UK data, in some infections, this duration can exceed 60 days. Although this may only represent a small proportion of the population (0.1 to 0.5 %), it might be compensated by the length of the transmission period (at least 6 times that of a traditional generation time). Such long infections are not present in the cohort analysed but could likely be included using cohorts that are more representative of the general population (in fact, a sensitivity analysis would be required to show that the exact shape of the virus kinetics matters on the result).

1.2 The temporal component of the model seems very limited. For example, the authors assume that the generation time (i.e. the time between two infections) is constant, which is at odds with the evolution of the Alpha variant or behavioural changes. In the Discussion, they specify that this is why this model was run only over 2020. This seems to be at odds with Figures 1, 3, and 4. If the authors wish to restrict their analysis to 2020, they should do it consistently throughout the manuscript. Conversely, if they analyse results up to mid-2021, they need to account for these temporal variations.

1.3 The authors write that they use an "individual-based" model but, unless I am mistaken, the individuals are not tracked. More precisely, the host population does not vary (there is no immunisation through natural immunity or vaccination). This is not an issue for the first time periods of course, but since the authors show results for mid-2021, although the cumulative incidence was not that high, it could have a disproportionate effect if one assumes that more connected individuals are more likely to be exposed (in which case they would also be more likely to be immune).

1.4 I was happy that the authors introduced confidence intervals for their plots on number of contacts in Figure 1. However, I am not sure that the binomial confidence intervals bring a lot. Another option, which we used with one of these contact datasets, could be to discretise the data (e.g. per week) and use these weekly values to generate a distribution of heterogeneity values. This would have the advantage to illustrate how variable the polls are from one week to the next.

1.5 A large part of the focus of the manuscript is devoted to the implementation of self-testing but it was unclear to me how this was modelled exactly (there are only 4 lines in the main text and none in the Supplementary materials). Also, the link between testing and symptoms is not mentioned (I could only find a discussion regarding self-isolation and symptoms). Finally, I think it would be interesting to show whether the implementation of self-testing has a different effect in a model without heterogeneity compared to a model with heterogeneity (in a scenario that is not a lock-down of course). This would help better demonstrate the importance of the current formalism.

2) References

One of the reasons why I stopped modelling COVID (besides the lack of funding), is that the bibliography changes at a huge pace. Here, given the public health focus of the article, I was expecting to read about some of the recent developments in the field. Unfortunately, among the 32 references, only 3 are from 2023 and 2 from 2024. This would of course not be a problem, even for such a rapidly moving field as the COVID one, if it only interfered with the introduction and discussion. However, I think this also impacts the methods and results.

2.1 I find the data used for the virus load kinetics a bit suboptimal, especially since in the end the authors use distributions with 3 parameters. The data originates from an NBA cohort where most of the individual tested are in excellent physical shape. Since then, new data has been released that seems essential to address the question here, especially data on long infections (e.g. see the work from Ghafari et al. 2024 Nature https://doi.org/10.1038/s41586-024-07029-4 ).

2.2 The link between SARS-CoV-2 viral load and contagiousness is tricky to calibrate. The authors lean on a 2021 study based on the probability of culturing the virus but, as for the infection duration, there is more robust work linking virus load and transmission (see e.g. Marc et al. 2021 eLife https://doi.org/10.7554/eLife.69302 )

2.3 I was wondering how the results from the UK app. showing that contact duration matters more than the exact distance could affect the empirical fitting of the data. In particular, people could have more contacts than they expect if distance is less of an issue (Ferretti et al. 2023 Nature https://doi.org/10.1038/s41586-023-06952-2 ). More generally, discussing the usefulness of a contact app. to estimate Rt, e.g. the study by Kendall et al. 2024 Science https://doi.org/10.1126/science.adm8103 ) seems relevant in this context.

2.4 Should the authors decide to keep their results from 2021, there is also some literature about how temporal variations in Rt heterogeneity could be explained by spatial structure and the depletion of susceptible hosts (Thomine et al. 2021 eLife https://doi.org/10.7554/eLife.71417 ). There could also be an effect of variations in generation time (e.g. Blanquart et al. 2021 eLife https://doi.org/10.7554/eLife.75791 ).

3) Fit with the journal

3.1 The manuscript was reviewed by PLoS Biology and probably sent directly to PLoS Computational Biology without any modification of the format. However, even when bearing this in mind, PLoS Comput Biol may not be the best fit. The main reason is that the insights of the work will clearly appeal to public health experts rather than to computational biologists. Even I feel poorly qualified to assess the public health impact of this work, e.g. compared to earlier results regarding the effect of self testing, and I think this should be done by a more specialised journal.

3.2 Related to the previous point, the strength of this work comes from the fact that the authors sample from empirical distributions (although questions can be raised about the dataset used for the virus load and about the variability of the one use for the contact data). This comes at a cost in terms of generalisation. I am unsure these results could be extended to other infections. Furthermore, does the use of empirical distributions (especially for virus load) yield different result than other families of functions consistent with the infectious duration of respiratory viruses? Again, with in a public health-oriented journal, this focused aspect of the research would be less of an issue.

3.3 Writing this piece for a less computational journal would also allow the authors to go into further details regarding the drivers of heterogeneity. I only glanced at the two articles they cite but neither of these seem to investigate these individuals with high number of contacts. However, they focus a lot on other criteria such as age. It would be interesting to see if these individuals with high number of contacts differ from the rest of the population because it would clearly impact the possibility to control the epidemic.

Detailed comments

line 252-254: Since Figure 3 was simulated using the data shown in Figure 1, isn't it expected that they look alike?

line 407: It might be interesting to compare the negative binomial distribution to others (line 407).

Table 1 seems redundant with Figure 1.

[signed and dated]

- Samuel Alizon, Nov 1, 2025

Reviewer #2: # SUMMARY

This analysis aims to quantify the relative influence of variability in daily contacts versus viral shedding on heterogeneity in onward transmission for SARS-CoV-2 using early pandemic contact data from the UK. Overall, it is an interesting question and the overall approach comparing 4 main scenarios seems reasonable. Some methods details were not clear enough, and some conclusions are a bit strong given the analytic framework. However, my main concerns are how correlations in contact data and durations are implemented, which does not make best use of the available data to answer this question.

# MAJOR ISSUES

1. **Contact correlations:** The methods note that household (HH) contacts were fixed after one random assignment, while non-HH contacts were sampled randomly each day. This makes sense to me for HH, but it's not clear from which distribution non-HH contacts were sampled. Did this sampling maintain correlations in a) individual-level contacts over time (e.g. some individuals consistently reporting more contacts than others), and b) durations of contacts (e.g. durations are likely shorter for individuals with many contacts).

1.a) I reviewed the results, methods, appendix, and github code, and I'm still unclear what's been done. From the code I think these correlations are ignored:

> https://github.com/bquilty25/superspreading_testing/blob/master/scripts/utils.R#L460

1.b) Using the UK CoMix data, I estimated that at least half of the variance in non-HH contacts is essentially explained by individual-level effects (versus time effects or random variations) [code below]. While reported contacts in CoMix do not reflect subsequent days as in the current analysis, this correlation still suggests non-negligible individual-level effects.

1.c) I expect that correct accounting of within-person correlation in contact daily non-HH contact numbers would drive even greater overdispersion in transmission due to contact heterogeneity, while accounting for duration correlations would have the opposite effect. It's not clear if these competing biases are of similar magnitude. Regardless, both correlations should be accounted for here, given the centrality of quantifying overdispersion to the research question, and it should be possible given the available data.

1.d) A follow-on issue is that the current analysis ignores the influence of contact heterogeneity on *acquisition* risk. This is briefly mentioned in the limitations [324]. If individuals' contacts are correlated over time, we would not expect the distribution of contacts among infected individuals (at risk of transmission) to match the population-level contact distribution (estimated in surveys) -- at least prior to widespread immunity. Correct accounting of this aspect would likely increase R(t) substantially, though the implications for overdispersion aren't obvious to me. Again, it should be possible to examine this given the available data.

2. **Contact durations:** While I appreciate the authors' efforts to account for contact durations, I am concerned about the implementation: dur_{(N)HH,i,j,t} are "defined as the proportion of a 24-hour period spent at home or outside of the home respectively that the contact lasted". So, if someone spends 16/24 hours at home and 8/24 hours outside, and they report two 1-hour contacts: 1 at home and 1 outside, the household contact would then have "dur" = 1/16 while the outside contact would have "dur" 1/8, making outside contacts (in this case) "doubly infectious". More generally, the weight would be inversely proportional to the time spent in one or another context.

# MEDIUM ISSUES

1. **Structure:** As with other similarly structured papers, the methods-last structure (suggested by the journal) feels inefficient and sometimes confusing. Various methods details are necessarily given in the results section, but these are inevitably repeated with some added detail in the final methods section, or with still more details sometimes in the appendix. I was never quite sure where to expect the complete details of any analysis step during first/second read. Some aspects aren't actually mentioned in the methods, such as pre-event testing.

2. **Overdispersion parameter:** It's quite counterintuitive that the chosen overdispersion parameter (k) decreases with variance. It's helpful this has been stated explicitly in a few places, but the reader must constantly be reminded of this counterintuitive relationship. Why not simply use the reciprocal \alpha = 1/k instead, as is sometimes done elsewhere? Or perhaps the coefficient of variation (SD/mean). It would be much easier to follow, throughout.

2.a) This would also help with the disappearing "all-homogeneous" case in Figure 4.

3. **Falsifiability:** I am inclined to agree with previous reviewers that it would be difficult to draw different conclusions (e.g. that VL variation actually is drives super-spreading more than contact heterogeneity) based on the present analysis.

3.a) Perhaps if the (presumably) negative correlation between daily contact number and durations were captured, then this would allow VL heterogeneity to exert more influence in those contexts with super-spreading potential.

# MINOR ISSUES

- [55+] I appreciate the authors' effort to succinctly label the two competing hypotheses here as "wrong-person" versus "wrong-time". However, the word "wrong" feels ... wrong here, and possibly vaguely stigmatizing (drawing on HIV/mpox work). Perhaps something like "between-person (variability)" versus "within-person variability" could better capture the idea?

- [100] Figure 1a: overall, this is a great illustration of these trends. Can a sequential colourmap be used here to make the trend across contact numbers more intuitive?

- [112] Table 1: is "N" here the number of unique respondents or survey responses? I assumed the latter, but it may be worth clarifying in a table note - and likewise for the (%) columns.

- [142] I had to re-read this very long sentence several times to understand everything being said: "By mapping viral load to infectivity via a logistic function based on viral load and culture (representing live, infectious virus) from Pickering et al. 8 (Figure 2B), calculating P(infectivity) by day (Figure 2C), then integrating under the infectivity curve, we can reproduce substantial heterogeneity in individual infectiousness as reported by Ke et al.6, with a >70-fold difference in individual-level infectivity between the 2.5% and 97.5% percentiles of the individual-level distribution (0.08 and 5.74, a.u, respectively) and a shape parameter of a fitted Gamma distribution of 1.42." Please consider breaking this up into shorter, clearer sentences. For example, is gamma distribution fitted to the density of relative infectivity over time? This is not clear, even after reviewing the corresponding methods text.

- [148] "When simulating whether or not individuals would infect a single contact on each day of their infection ..." I was confused reading this: have contact rates analyzed in the prior section now been incorporated? If so, for which time period?

- [165] I found the term "individual-based model" a bit misleading, as I was expecting a model that captured iterative acquisition/transmission chains, which is usually what "IBM" means in ID modelling. I recommend to use a different term, perhaps simply "stochastic model".

- [243] The statement "closely matching contemporaneous estimates" is a bit strong, given that all the R(t) estimates hover around 1.

- [248+] "dispersion in the reproduction number" is a bit strange to say, since the reproduction number is defined as the *mean* number of secondary cases per index case. Maybe "dispersion in onward transmission"?

- [267] I do not follow the logic of: 'superspreading is more a case of "wrong place, wrong time" than "wrong person".'

- [351] "other respiratory viruses that exhibit heterogeneous transmission, including influenza, RSV, and adenoviruses" can a citation be given here?

- [440] Can the authors clarify the maximum number of days post-exposure that were considered for onward transmission? Relatedly, the actual process of iterating over each day for each individual is only implied when the Bernoulli sampling is described, but this iteration could be stated more plainly.

- [github] The "utils.r" contains many steps of analysis code that I was surprised to find in a file called "utils"

# CODE

Here is code I used to estimate the variance explained by individual-level effects in CoMix UK:

```R

X = merge(read.csv('CoMix_uk_contact_common.csv'),

read.csv('CoMix_uk_participant_extra.csv'))

X = cbind(n=1,subset(X,!cnt_home)) # nhh contacts

A = aggregate(n~survey_round+panel_id,cbind(n=1,X),sum)

names(A) = c('t','id','n')

# complete dataset is too big: ah hoc bootstrap

S = do.call(rbind,parallel::mclapply(1:1000,function(i){

set.seed(i); ll = logLik

A.i = subset(A,id %in% sample(A$id,100,rep=0))

A.i[1:2] = lapply(A.i[1:2],as.factor)

m0 = glm(n~1, 'poisson',A.i)

mt = glm(n~t, 'poisson',A.i)

mi = glm(n~id, 'poisson',A.i)

mit = glm(n~id+t,'poisson',A.i)

S.i = data.frame(

d0=m0$dev,dt=mt$dev,di=mi$dev,dit=mit$dev,

l0=ll(m0),lt=ll(mt),li=ll(mi),lit=ll(mit))

},mc.cores=7))

# a few pseudo-R2 options ...

summary(1-S$dit/S$dt) # R^2_L (a) ~ 0.79

summary(1-S$di /S$d0) # R^2_L (b) ~ 0.68

summary(1-S$lit/S$lt) # R^2_CS (a) ~ 0.64

summary(1-S$li /S$l0) # R^2_CS (b) ~ 0.59

```

Reviewer #3: In the manuscript by Quilty et al., the authors combine previously published survey data and previously published within host virus data to model COVID-19 dynamics. Their particular focus is on measuring heterogeneity in transmission (i.e. superspreaders), and whether superspreading is more closely tied to contact rate or infectiousness. They conclude that contact rate, as opposed to infectiousness, is the key driver of heterogeneity. They go on to talk about testing rates and lockdowns and the influence these things had (or could have) on disease dynamics. Overall, I thought this was a very interesting manuscript, but I have numerous suggestions for improvement.

First, I think the authors should take some time to elaborate on a conceptual issue with these types of “which is more important A or B?” type studies. That being, if one variable is measured more accurately than the other, the more accurately estimated thing will appear more important even if it isn’t. In this case, the hosts measure of “contact rate” is a direct measure of contact rate, whereas virus load within hosts is a proxy for infectiousness. There are sources of variation in infectiousness that may be strongly decoupled from within-host dynamics, which could greatly increase the variation in transmissibility attributable to infectiousness. These things might include innate factors (propensity to cough, antibody cross-reactivity) or behavioral factors distinct from contact related things (i.e. wiping nose vs. blowing, how close you stand to people, how loudly you talk). As the authors say in lines 422-425, Ke found heterogeneity in infectiousness that extended beyond the within host dynamics, and so the decision to leave it out potentially biases their results to find that contact rate is proportionally more important than it should be and infectiousness is proportionally less important.

Another important point that the authors don’t currently discuss is biases/limitations in their survey data that could lead to “fake” overdispersion. I am certainly not an expert with these types of data, but I know a bit about them. A challenge that I have seen with contact tracking data is that different people might report the same event differently. For example, two people go to dinner with each other. One might say that they had one contact, and the other might say that they had 50 contacts (i.e. number of patrons in the restaurant). Of course, in practice their effective number of contacts is the same, and somewhere between one and 50. It would be nice to hear a little more about this survey and how they did or did not control for this issue. Of course, there is no perfect solution and the impact of this should be discussed. Just using this example as an illustration, the authors’ model would predict that there was drastic overdisperion, with one of the individuals being a superspreader and the other not even though the only difference here is in the reporting.

There were also a few places (particularly the first two paragraphs of the discussion) where the conclusions of the models seemed overstated. On lines 242-243 the authors state that they were able to reconstruct the secondary infection distributions from first principles. In reality, they showed that they were able to reconstruct the “mean” number the secondary infections, which is seems like a very different statement to me. Unless I missed it, Fig 3 shows they are doing a reasonable job on the mean, but does not include real data on overdispersion. Also, on lines 252-255, “Our analysis shows that dynamic shifts in the mean and overdispersion of the reproductive number closely track the changes in the mean and overdispersion of daily number of contacts,” but I think this is an overstatement of their results. Really what they are doing here is showing that the model predicted overdispersion matches the overdispersion in their contact rate dataset (which it should since this is essentially a parameter in the model). The next sentence is equally an overstatement of the results, “… contact overdispersion can be reliably measured in contact surveys and is predictive of superspreading.” As far as I can tell, saying it is “reliable” or “predictive” is not justified since there is no ground-truth data used in this manuscript to compare against. Likewise, the authors say (lines 263-264) that “>50% estimated to have viral loads capable of infecting many others on at least one day of their infection”. As far as I can tell, this statement is highly dependent on a model assumption that infectiousness can be equated with the probability of culturing virus. Had they made a different assumption here, that 50% number could presumably be drastically different.

Smaller points:

- As said earlier, sometimes the authors had a propensity to overstate their results, but in other places, they sort of seem to miss what I would consider to be the most interesting bits. Lines 28-30, the authors talk about testing every day to reduce the reproductive number. This feels sort of obvious and selling short the punch of the paper. I think it is because of the "test every 3 days bit". I feel like it would be a lot more interesting to reframe it as, "having everyone in the population test every x days would be equivalent to having people test only before events with minimum size y". To me, that sounds punchier, and it also highlights the main advance of the paper, while providing a clear use-case for this type of modeling approach.

- I realize that the format for this journal is to have the methods at the end, but around line 165, I felt like I needed to know a little bit about what the model looked like before I could understand/believe the text. It might be possible to put in a short (e.g. 1-3 sentence) description here.

- Lines 216-219. This result seems confounded with the functional relationship between load and test detection rate. It would be cleaner to potentially remove the assumption of a correlation here and instead say about how the effect would be even stronger if there was a relationship between within host load and test detection rate.

- Line 249-250. The authors talk about the degree of overdispersion as being between 0.3 and 0.6 but do not provide units for interpretation. Is this meant to be “k”? Also, referring to “k” as overdispersion (such as in Fig 4) is confusing because overdispersion is greatest when k is small. Better would be to say that k is an inverse measure of overdispersion. Most accurately though, I would say that overdispersion is 1/sqrt(k), i.e. the coefficient of variation.

- Line 269. I’m either confused or there is a typo. The authors say that interventions can be targeted at individuals during their period of high infectivity by testing their contacts, but this would presumably be a delayed response that would be useless because you would only know that someone was a superspreader after they left their highly infectious period.

- Figure 6, I don’t understand why one contact of Individual C seems to become infected and then revert back to non-infected. Maybe I am misinterpreting this figure?

- Lines 452 and 453, the authors use the abbreviation LFTs here, which doesn’t seem necessary since they made it through 95+% of the manuscript without needing it. I found myself having think pretty hard to figure out what they meant by it.

**********

**Have the authors made all data and (if applicable) computational code underlying the findings in their manuscript fully available?**

The PLOS Data policy requires authors to make all data and code underlying the findings described in their manuscript fully available without restriction, with rare exception (please refer to the Data Availability Statement in the manuscript PDF file). The data and code should be provided as part of the manuscript or its supporting information, or deposited to a public repository. For example, in addition to summary statistics, the data points behind means, medians and variance measures should be available. If there are restrictions on publicly sharing data or code —e.g. participant privacy or use of data from a third party—those must be specified.

Reviewer #1: Yes

Reviewer #2: None

Reviewer #3: None

**********

PLOS authors have the option to publish the peer review history of their article (what does this mean?). If published, this will include your full peer review and any attached files.

If you choose “no”, your identity will remain anonymous but your review may still be made public.

**Do you want your identity to be public for this peer review?**  For information about this choice, including consent withdrawal, please see our Privacy Policy.

Reviewer #1: **Yes:** Samuel Alizon

Reviewer #2: No

Reviewer #3: No

[NOTE: If reviewer comments were submitted as an attachment file, they will be attached to this email and accessible via the submission site. Please log into your account, locate the manuscript record, and check for the action link "View Attachments". If this link does not appear, there are no attachment files.]

**Figure resubmission:**

**Reproducibility:**

?>

# Author comment: Author response to Decision Letter 1

**DOI:** 10.1371/journal.pcbi.1014715.r002

29 Apr 2026

Attachment Submitted filename: Response_to_reviewers.pdf

# Peer review: Decision Letter 1

**DOI:** 10.1371/journal.pcbi.1014715.r003

**Authors:** Nicholas Geard, Benjamin Althouse

27 May 2026

PCOMPBIOL-D-25-01964R1

Disentangling the drivers of heterogeneity in SARS-CoV-2 transmission from data on viral load and daily contact rates

PLOS Computational Biology

Dear Dr. Chapman,

Thank you for submitting your manuscript to PLOS Computational Biology. After careful consideration, we feel that it has merit but does not fully meet PLOS Computational Biology's publication criteria as it currently stands. Therefore, we invite you to submit a revised version of the manuscript that addresses the points raised during the review process.

Please submit your revised manuscript by Jul 27 2026 11:59PM. If you will need more time than this to complete your revisions, please reply to this message or contact the journal office at ploscompbiol@plos.org. When you're ready to submit your revision, log on to https://www.editorialmanager.com/pcompbiol/ and select the 'Submissions Needing Revision' folder to locate your manuscript file.

Please include the following items when submitting your revised manuscript:

* A letter that responds to each point raised by the editor and reviewer(s). You should upload this letter as a separate file labeled 'Response to Reviewers'. This file does not need to include responses to formatting updates and technical items listed in the 'Journal Requirements' section below.

* A marked-up copy of your manuscript that highlights changes made to the original version. You should upload this as a separate file labeled 'Revised Manuscript with Track Changes'.

* An unmarked version of your revised paper without tracked changes. You should upload this as a separate file labeled 'Manuscript'.

If you would like to make changes to your financial disclosure, competing interests statement, or data availability statement, please make these updates within the submission form at the time of resubmission. Guidelines for resubmitting your figure files are available below the reviewer comments at the end of this letter.

As the corresponding author, your ORCID iD is verified in the submission system and will appear in the published article. PLOS supports the use of ORCID, and we encourage all coauthors to register for an ORCID iD and use it as well. Please encourage your coauthors to verify their ORCID iD within the submission system before final acceptance, as unverified ORCID iDs will not appear in the published article. Only the individual author can complete the verification step; PLOS staff cannot verify ORCID iDs on behalf of authors.

We look forward to receiving your revised manuscript.

Kind regards,

Nicholas Geard

Academic Editor

PLOS Computational Biology

Benjamin Althouse

Section Editor

PLOS Computational Biology

**Additional Editor Comments:**

Thank you for your detailed response and revision. The reviewers are in agreement that the message and methods are much clearer, and the additional sensitivity analyses increase confidence in the robustness of your results. They have identified a small number of minor issues which should, I think, be straightforward to address, and made several suggestions regarding scope, model choices, and uncertainty, which I encourage you to consider.

**Reviewers' comments:**

Reviewer's Responses to Questions

**Comments to the Authors:**

**Please note here if the review is uploaded as an attachment.**

Reviewer #1: I thank the authors for considering my feedbacks. I now find that the key message (heterogeneity in contacts matter more than that in virus load regarding epidemic spread) is much clearer. However, I still have a few issues.

1) Clarifying the scope. From the title, the authors insist on their use of "data on viral load and daily contacts". However, for the virus load at least, what the really reuse are estimates. This could be clarified in the abstract and in the main text. The fact that this data is already published could also be stressed.

2) The sociodemographic characteristics of the data used is still not presented in details. This is I think a major issue because these might explain some differences, e.g. on the pre- and the first post-pandemic period, if the participants to the BBC experiment are wealthiers or younger.

3) The authors decided to resimulate the individual kinetics data from the estimates found by Kissler et al. whereas for the contact data they use individual trajectories. If taking the individual Ct trajectories is not feasible because of the truncations, why not take the individual posteriors?

4) Several references are off (they all point to Ref. 8 instead of the correct one). Furthermore, several figures (e.g. Figs 2C, 3D, or 6) are missing their confidence interval (or the caption is missing a description of what the shaded area exactly stands for).

5) In the discussion, it could be worth extending the scope since this question of the importance of infectiousness vs. number of contacts can apply to any infectious disease.

Detailed comments

l.72-73: this is an assumption and should be presented as such.

l.88: Are kids allows to use their apps at school in the UK?

l.125: please remind the reader the exact dates.

l.133, 146c: wrong references

l.140: This is data from synthetic T7 RNA transcripts. Is it the most appropriate?

l.195: How is beta set?

l.322-323: the infectiouness duration in this model seems very low compared to the serial intervial estimates.

l.388: Perhaps also show the results with the doubled variance on this figure?

[signed and dated]

- Samuel Alizon, 19 May 2026

Reviewer #2: Overall I am satisfied with the additional changes, in particular: the methods are much clearer now, and the added sensitivity analyses help ensure the headline finding is robust --- that contact heterogeneity likely plays a larger role than viral shedding heterogeneity in overdispersion.

A few additional minor points, which I leave to the editors / authors to decide if they are necessary to address in another round of revisions.

+ The methods section [108] "analysis of social contact data" and corresponding results section [257] "changes in the distribution of social contacts with changes in the intensity of restrictions" are not really introduced as an objective -- essentially a descriptive analysis of the data. While these are useful for the paper, they currently feel unexpected when reading on first pass based on the current introduction.

+ [223] It seems like we are missing a brief description about how the homogeneous viral load trajectory case is defined - presumably applying the mean parameters to everybody...

+ [225] It would be better to use the word "sensitivity analysis" in the first sentence of this paragraph, so it's clear we are deviating from the main analysis.

+ [600] I appreciate the added discussion of heterogeneous acquisition risk here. I might even add that, if anything, heterogeneity in acquisition risk would likely further amplify the relative importance of contact heterogeneity (versus heterogeneity in viral load trajectories), even under homogeneous mixing, and even more under homophilic mixing. Also, I am not really satisfied with the justification in the letter [R2.1.d] to avoid weighting acquisition risk by contact rates because it requires "additional assumptions" or data, since the current implementation makes arguably a stronger assumption implicitly, also without evidence: that acquisition risk is independent of contact rate. Nevertheless, leaving this as a limitation is acceptable to me.

+ [Figure 5] It's a hard to see the dotted vs solid line in the legend/guide, and the CI are hard to distinguish in the top panel. Why not use 3 distinct colours instead?

Reviewer #3: I am mostly happy with the revisions. One slightly bigger point and one smaller point remain.

The bigger point: The authors really want to say that a large fraction of individuals were infectious at some point in their infections, but I don't think they can justify this statement. The statement is built on this line: "culture positivity is the best available correlate of the infectious period, outperforming both PCR and symptom monitoring (Drain et al.);", but I looked into that manuscript and the statement is not justified by the publication. Drain et al. just assume that hosts are infectious when cultures are positive -- this is not based on any data in their manuscript. It also isn't biologically justifiable given that culture positivity (they aren't measuring TCID50, just yes/no) will depend on how much sample is used on each plate. As best I can tell, Drain et al. literally replace the term "culture positivity" with "infectiousness" and then look for other correlates of "infectiousness". This means that the conclusions in the present manuscript about the fraction of individuals infectious at various points in time are based on the faulty premise that culture positivity equates to infectiousness. Growth in culture is probably related to infectiousness, but the threshold for where something should be called infectious doesn't seem to be known (or at least, not from Drain et al.). This should at minimum be discussed as a limitation of the study. I get that the authors have done some sensitivity analyses, and so this doesn't seem like a fatal flaw, but I would encourage the authors to acknowledge the uncertainty.

Smaller point: The authors say in a few places (most notably the abstract and second discussion paragraph) that you can "predict" superspreading with contact rate data, but this is a strange choice of words, because (as the authors argue) the superspreading event is the heterogeneity in the contact rate data. And you can't predict something that has already happened.

**********

**Have the authors made all data and (if applicable) computational code underlying the findings in their manuscript fully available?**

The PLOS Data policy requires authors to make all data and code underlying the findings described in their manuscript fully available without restriction, with rare exception (please refer to the Data Availability Statement in the manuscript PDF file). The data and code should be provided as part of the manuscript or its supporting information, or deposited to a public repository. For example, in addition to summary statistics, the data points behind means, medians and variance measures should be available. If there are restrictions on publicly sharing data or code —e.g. participant privacy or use of data from a third party—those must be specified.

Reviewer #1: None

Reviewer #2: None

Reviewer #3: None

**********

PLOS authors have the option to publish the peer review history of their article (what does this mean?). If published, this will include your full peer review and any attached files.

If you choose “no”, your identity will remain anonymous but your review may still be made public.

**Do you want your identity to be public for this peer review?**  For information about this choice, including consent withdrawal, please see our Privacy Policy.

Reviewer #1: **Yes:** Samuel Alizon

Reviewer #2: No

Reviewer #3: No

[NOTE: If reviewer comments were submitted as an attachment file, they will be attached to this email and accessible via the submission site. Please log into your account, locate the manuscript record, and check for the action link "View Attachments". If this link does not appear, there are no attachment files.]

**Figure resubmission:**

**Reproducibility:**

To enhance the reproducibility of your results, we recommend that authors of applicable studies deposit laboratory protocols in protocols.io, where a protocol can be assigned its own identifier (DOI) such that it can be cited independently in the future. Additionally, PLOS ONE offers an option to publish peer-reviewed clinical study protocols. Read more information on sharing protocols at https://plos.org/protocols?utm_medium=editorial-email&utm_source=authorletters&utm_campaign=protocols

# Author comment: Author response to Decision Letter 2

**DOI:** 10.1371/journal.pcbi.1014715.r004

16 Jul 2026

Attachment Submitted filename: Response to reviewers.docx

# Editor report: Decision Letter 2

**DOI:** 10.1371/journal.pcbi.1014715.r005

**Authors:** Nicholas Geard, Benjamin Althouse

13 Aug 2026

Dear Dr Chapman,

We are pleased to inform you that your manuscript 'Disentangling the drivers of heterogeneity in SARS-CoV-2 transmission from data on viral load and daily contact rates' has been provisionally accepted for publication in PLOS Computational Biology.

Before your manuscript can be formally accepted you will need to complete some formatting changes, which you will receive in a follow up email. A member of our team will be in touch with a set of requests.

Please note that your manuscript will not be scheduled for publication until you have made the required changes, so a swift response is appreciated.

IMPORTANT: The editorial review process is now complete. PLOS will only permit corrections to spelling, formatting or significant scientific errors from this point onwards. Requests for major changes, or any which affect the scientific understanding of your work, will cause delays to the publication date of your manuscript.

Should you, your institution's press office or the journal office choose to press release your paper, you will automatically be opted out of early publication. We ask that you notify us now if you or your institution is planning to press release the article. All press must be co-ordinated with PLOS.

Thank you again for supporting Open Access publishing; we are looking forward to publishing your work in PLOS Computational Biology.

Best regards,

Nicholas Geard

Academic Editor

PLOS Computational Biology

Benjamin Althouse

Section Editor

PLOS Computational Biology

***********************************************************

# Editor report: Acceptance letter

**DOI:** 10.1371/journal.pcbi.1014715.r006

**Authors:** Nicholas Geard, Benjamin Althouse

PCOMPBIOL-D-25-01964R2

Disentangling the drivers of heterogeneity in SARS-CoV-2 transmission from data on viral load and daily contact rates

Dear Dr Chapman,

I am pleased to inform you that your manuscript has been formally accepted for publication in PLOS Computational Biology. Your manuscript is now with our production department and you will be notified of the publication date in due course.

The corresponding author will soon be receiving a typeset proof for review, to ensure errors have not been introduced during production. Please review the PDF proof of your manuscript carefully, as this is the last chance to correct any errors. Please note that major changes, or those which affect the scientific understanding of the work, will likely cause delays to the publication date of your manuscript.

Soon after your final files are uploaded, unless you have opted out, the early version of your manuscript will be published online. The date of the early version will be your article's publication date. The final article will be published to the same URL, and all versions of the paper will be accessible to readers.

For Research, Software, and Methods articles, you will receive an invoice from PLOS for your publication fee after your manuscript has reached the completed accept phase. If you receive an email requesting payment before acceptance or for any other service, this may be a phishing scheme. Learn how to identify phishing emails and protect your accounts at https://explore.plos.org/phishing.

Thank you again for supporting PLOS Computational Biology and open-access publishing. We are looking forward to publishing your work!

With kind regards,

Sharmila Kamatchi

PLOS Computational Biology | Carlyle House, Carlyle Road, Cambridge CB4 3DN | United Kingdom ploscompbiol@plos.org | Phone +44 (0) 1223-442824 | ploscompbiol.org | @PLOSCompBiol
